# DDAH-1 maintains endoplasmic reticulum-mitochondria contacts and protects dopaminergic neurons in Parkinson’s disease

**DOI:** 10.1038/s41419-024-06772-w

**Published:** 2024-06-07

**Authors:** Yichen Zhao, Weiwei Shen, Minjie Zhang, Min Guo, Yunxiao Dou, Sida Han, Jintai Yu, Mei Cui, Yanxin Zhao

**Affiliations:** 1 Department of Neurology, Shanghai Tenth People’s Hospital, Tongji University School of Medicine, Shanghai, China; 2 Department of Neurology and Institute of Neurology, Huashan Hospital, Shanghai Medical College, Fudan University, Shanghai, China; 3 Department of Neurology, Huashan Hospital, MOE Frontiers Center for Brain Science, Fudan University, Shanghai, China

**Keywords:** Parkinson's disease, Experimental models of disease

## Abstract

The loss of dopaminergic neurons in the substantia nigra is a hallmark of pathology in Parkinson’s disease (PD). Dimethylarginine dimethylaminohydrolase-1 (DDAH-1) is the critical enzyme responsible for the degradation of asymmetric dimethylarginine (ADMA) which inhibits nitric oxide (NO) synthase and has been implicated in neurodegeneration. Mitochondrial dysfunction, particularly in the mitochondria-associated endoplasmic reticulum membrane (MAM), plays a critical role in this process, although the specific molecular target has not yet been determined. This study aims to examine the involvement of DDAH-1 in the nigrostriatal dopaminergic pathway and PD pathogenesis. The distribution of DDAH-1 in the brain and its colocalization with dopaminergic neurons were observed. The loss of dopaminergic neurons and aggravated locomotor disability after rotenone (ROT) injection were showed in the DDAH-1 knockout rat. l-arginine (ARG) and NO donors were employed to elucidate the role of NO respectively. In vitro, we investigated the effects of DDAH-1 knockdown or overexpression on cell viability and mitochondrial functions, as well as modulation of ADMA/NO levels using ADMA or ARG. MAM formation was assessed by the Mitofusin2 oligomerization and the mitochondrial ubiquitin ligase (MITOL) phosphorylation. We found that DDAH-1 downregulation resulted in enhanced cell death and mitochondrial dysfunctions, accompanied by elevated ADMA and reduced NO levels. However, the recovered NO level after the ARG supplement failed to exhibit a protective effect on mitochondrial functions and partially restored cell viability. DDAH-1 overexpression prevented ROT toxicity, while ADMA treatment attenuated these protective effects. The declines of MAM formation in ROT-treated cells were exacerbated by DDAH-1 downregulation via reduced MITOL phosphorylation, which was reversed by DDAH-1 overexpression. Together, the abundant expression of DDAH-1 in nigral dopaminergic neurons may exert neuroprotective effects by maintaining MAM formation and mitochondrial function probably via ADMA, indicating the therapeutic potential of targeting DDAH-1 for PD.

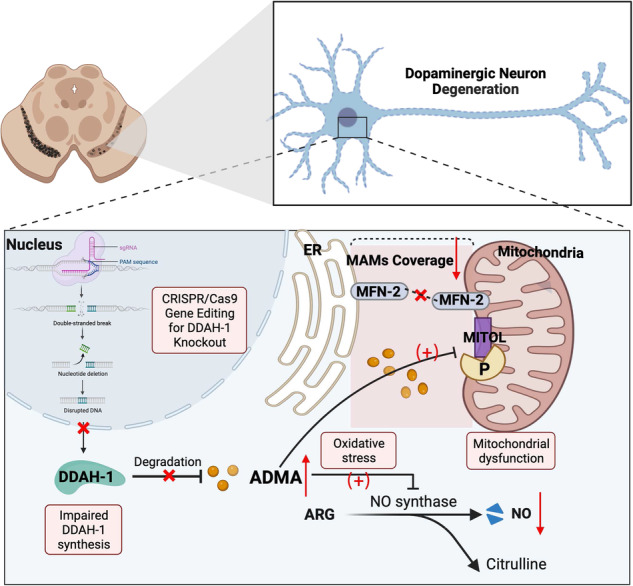

## Introduction

Parkinson’s disease (PD) is a movement disorder that primarily affects elderly [1] and has significant negative impacts on patients’ quality of life due to its motor and non-motor symptoms [2, 3]. Despite the primary pathology of PD being the loss of dopaminergic neurons in the substantia nigra pars compacta (SNpc), the exact mechanisms leading to this specific type of neuronal loss remain unclear [4].

Asymmetric dimethylarginine (ADMA) is a naturally occurring endogenous inhibitor of nitric oxide synthase (NOS), which competes with l-arginine (ARG) to bind to the active site of NOS and obstructs the generation of NO [5]. As a potential metabolite biomarker, ADMA has been recently linked to pathomechanistic pathways of neurodegeneration via regulation of NO levels [5]. Dimethylarginine dimethylaminohydrolase-1 (DDAH-1) is highly expressed in the brain and co-localized with neurons, regarded as the primary isoform responsible for metabolizing ADMA [6]. DDAH-1 is protective in ischemic stroke and enhances ischemic tolerance in hypoxic preconditioning [7, 8]. However, its distribution in the nigrostriatal dopaminergic pathway and its role in PD pathogenesis have not yet been reported.

Mitochondrial dysfunction, specifically within the mitochondria-associated endoplasmic reticulum membrane (MAM), is a well-established mechanism underlying dopaminergic dysfunction [9–11]. Mitofusin (MFN)2, a mitochondrial GTPase enriched in MAM, is a natural candidate to mediate the tethering of ER and mitochondria [12]. Mitochondrial ubiquitin ligase (MITOL) is reported to regulate the oligomerization and activation of MFN2 at MAM [13, 14]. However, it is still unknown whether certain proteins could mediate the activation of MFN2 or endoplasmic reticulum (ER)–mitochondrial tethering through MITOL under PD pathogenesis. Elevated ADMA levels have been associated with decreased mitochondrial membrane potential (MMP) and suppressed mitochondrial biogenesis, probably via inhibiting NO synthesis [15–17]. It remains to be investigated whether DDAH-1/ADMA exerts cellular regulatory effects via modulating NO metabolism in PD model.

Therefore, our study aimed to investigate the roles of DDAH-1 in rotenone (ROT) PD models in vivo and in vitro. We found that DDAH-1 was highly expressed within the dopaminergic neurons in substantia nigra (SN) and depletion of DDAH-1 aggravated the loss of dopaminergic neurons and the injury of locomotor ability of ROT-injected rats, which were reversed by ARG and NO donor. Our results also demonstrated that DDAH-1 played a protective role in ROT-induced neural death and mitochondrial dysfunction via ADMA. Mechanistically, DDAH-1 regulated MITOL phosphorylation and MFN2 oligomerization to maintain the MMP and ER-mitochondrial contacts probably by decreasing the ADMA level, which may be independent of NO pathway. This findings suggested the potential of DDAH-1 as a therapeutic target for PD.

## Material and methods

### Cell culture, treatment, and transfection

PC12 cell was purchased from Wuhan Sunncell Biotechnology Co., Ltd with STR profiling and tested for mycoplasma contamination, culturing in RPMI-1640 medium supplemented with 10% fetal bovine serum (FBS), 100 μg/mL streptomycin, and 100 U/mL penicillin G at 37 °C in 5% CO_2_. ROT (MCE, HY-B1756) and ADMA (MCE, HY-113216) were reconstituted in dimethyl sulfoxide (DMSO) while ARG (Sigma-Aldrich, A8094) was soluble in distilled water, prior to each experiment. The most suitable intervention condition including time and concentrations of ROT and ADMA were explored and the incubation of 0.1 μM ROT for 24 h and 80 μM ADMA for 24 h were chosen. 5 mM of ARG were treated as the supplement of NO for 24 h according to previous studies [18, 19]. PC12 cells were transfected with either DDAH-1 overexpressing or DDAH-1 small interfering RNA (siRNA) plasmid (Genomeditech, Shanghai) using Lipofectamine 3000 (Invitrogen, Carlsbad, USA), according to the manufacturer’s protocol for 48 h, followed by the treatment of ROT and ADMA or ARG for another 24 h, respectively. The overexpression and knockdown efficiency of DDAH-1 were assessed using reverse transcription-quantitative polymerase chain reaction (RT-qPCR) and western blot analyses.

### Cell viability measurement

Cell viability was assessed through the cell counting kit-8 (CCK-8, Beyotime, C0038) method. Briefly, CCK-8 solution was added to cells in 96-well plates. After incubation at 37 °C for 1 h, the optical density (OD) was measured by an absorbance microplate reader at 450 nm. The cell viability was expressed as a percentage of treated groups of the control group. Each sample was evaluated in triplicate.

### Reverse transcription-quantitative polymerase chain reaction

The mRNA levels of DDAH-1 in the transfected cells were analyzed using RT-qPCR. The total RNA was collected and the RNA concentration and purity were estimated using an ultraviolet spectrophotometer. The OD260/OD280 ratio was determined to be between 1.8 and 2.0. The RNA was reverse transcribed into cDNA using a PrimeScript RT reagent kit (Takara Bio Inc., Otsu, Japan), and the obtained cDNA was stored at −20 °C. The primers for glyceraldehyde-3-phosphate dehydrogenase (GAPDH), DDAH-1 of rats were designed and synthesized by Shanghai Sangon Biotechnology Co., Ltd (Shanghai, China). The primer sequences used were as follows: DDAH-1: 5′-CAGCCACCCCTCGGTCTT-3′ (forward), 5′-AAGCCGTCCACCTTTTCCAT-3′ (reverse); GAPDH: 5′-ACCACAGTCCATGCCATCAC-3′ (forward), 5′-TCCACCACCCTGTTGCTGTA-3′ (reverse). RT-qPCR was conducted with the cDNA template following the instructions of the SYBR FAST qPCR Master Mix kit (KAPA) by using an ABI7500 qPCR instrument (7900, Applied Biosystems, CA, USA), and GAPDH was used as an internal control. The 2^−ΔΔCt^ method was employed to calculate the expression level of mRNA. The experiment was repeated three times.

### Measurement of intracellular ADMA concentrations

The intracellular ADMA concentrations were measured by enzyme-linked immunosorbent assay (ELISA) kit (JianglaiBio, Shanghai, China) following the manufacturer’s instructions. After plasmid transfection and treatment of ROT and ADMA/ARG, each group of cells was collected and each sample was evaluated in triplicate. A standard curve of six standard concentrations and their corresponding OD values (450 nm) was generated to determine the concentration of the unknown sample.

### Determination of NO concentration

NO can be oxidized into nitrite and nitrate. The level of NO is determined by measuring the total amount of nitrite ion NO^2-^ and nitrate ionizer NO^3-^. The midbrain and PC12 cells were rapidly isolated, homogenized and lysated. After centrifugation of 3000 rpm for 6 min, the supernatant was collected for the determination of NO^2−^/NO^3−^ contents (μmol/gprotein or pg/mL) according to the manufacturer’s instruction of the NO assay kit (Nanjing Jiancheng Bioengineering Institute, China), which indirectly reflected NO concentration. Each sample was evaluated in triplicate.

### Measurement of MMP

A 5,5′,6,6′-tetrachloro-1,1′,3,3′-tetraethylbenzimidazolylcarbocyanide iodide (JC-1) probe (MCE, CBIC2) was used to measure the changes of inner MMP. At a high membrane potential, JC-1 accumulated in the mitochondria and formed red fluorescent aggregates, but existed mainly in the green fluorescent-monomeric form at low membrane potential. The cells were incubated with 2 μg/mL JC-1 for 30 min at 37 °C. After washing with PBS, the red and green fluorescence (red, λex/λem: 585/590 nm; green, λex/λem: 514/529 nm) was observed under a confocal microscope (Olympus, Tokyo, Japan). The fluorescence signal intensity was calculated using ImageJ software. The ratio of red/green fluorescence intensity was calculated to be an indicator of MMP. Mitochondrial depolarization was reflected by a decrease in the ratio. Each sample was evaluated in triplicate.

### Enzymatic assay for mitochondrial respiratory chain complex

The mitochondrial respiratory chain complex (MRCC) activity was measured using the Mitochondrial Complex Activity Assay Kit (Solarbio, China), according to the manufacturer’s protocol. Briefly, isolated mitochondrial pellets were suspended in hypotonic buffer and subjected to three freeze-thaw cycles. The concentration of mitochondrial proteins was measured using the bicinchoninic acid kit. Complex I activity was measured in the presence of decylubiquinone as the ROT-sensitive decrease in nicotinamide adenine dinucleotide at 340 nm. The absorbance was measured using a microplate reader (MD SpectraMax iD5, USA). Each sample was evaluated in triplicate.

### Mitochondrial calcium imaging

Cells were loaded with the fluorescent mitochondrial Ca^2+^ indicator Rhod2-AM (Abcam, ab142780, 1μm) following the manufacturer’s guidelines. Cells were washed three times with PBS and then were immediately observed using confocal microscope (Olympus, Tokyo, Japan). The average fluorescence intensity was calculated using ImageJ software and normalized to the cell number. Each sample was evaluated in triplicate.

### Detection of reactive oxygen species production

BBoxiProbe® dihydroethidium (DHE) reactive oxygen species (ROS) probe is oxidized to produce red fluorescent substance in the presence of in tissues, and the red fluorescence intensity is proportional to the level of ROS in tissues, followed by detecting the fluorescence intensity of DHE products at excitation wavelength 510 nm and emission wavelength 610 nm. To detect cellular ROS accumulation, the treated PC12 cells were seeded in a 24-well plate and loaded with DHE probe (Bestbio, BB-470515) and incubated at 37 °C for 30 min. The plates were then washed, and the fluorescence was measured using a microplate reader (MD SpectraMax iD5, USA). The values were normalized to the control and each sample was evaluated in triplicate.

### Transmission electron microscopy

Following the plasmid transfection and ROT treatment, the morphology of ER-mitochondrial contacts was observed by transmission electron microscopy (TEM). Cells in each group were collected and fixed in 2.5% (vol/vol) glutaraldehyde (pH 7.4) for in 0.1 M phosphate buffer at room temperature 2 h, embedded in agarose with low melting point, subsequently rinsed by 0.1 M phosphate buffer (pH 7.2) three times and fixed in 1% osmic acid at 4 °C for 2 h. Then the pallets were gradient dehydrated in a graded series of ethanol, embedded in Epon-Araldite resin for penetration and placed in a model for polymerization. Following the counterstaining of 3% uranyl acetate and 2.7% lead citrate, the ultrathin sections were prepared for examination via a HT7800 transmission electron microscope. Each sample was evaluated in triplicate.

### Isolation of mitochondria

The isolation of mitochondria from PC12 cells was performed using mitochondrial isolation kit (Thermo Fisher Scientific, USA) with the manufacturer’s instructions. The purified mitochondria were used for the following immunoblotting for MFN2 expression, blue native-PAGE for MFN2 oligomerization and assay for MRCC activity.

### Antibodies, immunoprecipitation, and immunoblotting

Antibodies obtained from the indicated suppliers were listed as followed: DDAH-1 (Abcam, ab180599, 1:5000), DDAH-2 (Abcam, ab184166, 1:1000), tyrosine hydroxylase (TH, Abcam, ab315207, 1:5000), MFN2 (Abcam, ab56889, 1:1000), cytochrome c oxidase IV (COX IV, Abcam, ab202554, 1:2000), β-actin (ACTB, Sigma, SAB1305546, 1:2000), MITOL (CST, 19168, 1:1000), phosphoserine (Abcam, ab9332, 1:250), horseradish peroxidase-conjugated secondary antibody (Abcam, ab6728, ab6721, 1:2000). Transfected and treated cells were harvested, and lysed in lysis buffer (50 mM Tris–HCl pH 8.0, 150 mM NaCl, and 0.1% SDS) for immunoblotting or in TNE buffer (50 mM Tris-HCl, pH 7.4 150 mM NaCl, 5 mM EDTA, 1% NP-40, 0.25% Na-deoxycholate, and 1 mM NaF) for immunoprecipitation/immunoblotting. Immunoprecipitation/immunoblot analyses were performed using standard protocols. Each sample was evaluated in triplicate. The specificity of anti-phosphoserine antibody to phosphorylated serine was confirmed by immunoblotting of MITOL extracted from transfected cells. The immunoreactive bands were scanned on Amersham Imager 600 using Enhanced Chemiluminescence Kit (Sangon Biotechnology, Shanghai, China), and the relative amounts of proteins were analyzed using ImageJ software.

### Blue native-PAGE

Mitochondria were purified as described above and 100‒200 μg mitochondria were lysed in blue native lysis buffer (20 mM HEPES pH7.4, 50 mM NaCl, 2.5 mM MgCl2, 10% glycerol, 0.1 mM EDTA, 0.5 mM PMSF, 1‒1.5% digitonin) at a concentration of 5 mg/ml for 30 min on ice. After a centrifugation at 21,000 × *g* for 10 min, the mitochondrial extract was separated on a 4‒14% blue native-PAGE. Proteins were transferred to a PVDF membrane and detected by immunoblot analysis using the indicated antibodies (MFN2, Abcam, ab56889, 1:1000). Each sample was evaluated in triplicate.

### Animals

DDAH-1 knockout (KO) (DDAH-1^-/-^) male Sprague–Dawley (SD) rats were a kind gift provided by Professor Da-Chun Xu (Department of Cardiology, Shanghai Tenth People’s Hospital, Tongji University School of Medicine). The CRISPR-Cas9 technique was applied to generate DDAH-1^-/-^ rats on SD background as illustrated in the supplementary material (Fig. S1a). Wild-type (WT) male SD rats (10 weeks of age, 300‒350 g) were purchased from Shanghai SIPPR-BK Laboratory Animal Co. Ltd and housed in separated cages in specific pathogen-free (SPF) animal room at 25 °C and humidity of 40% with a 12-h light/dark cycle. The healthy and weight/age-matched DDAH-1^-/-^ and WT rats were included in our study. The animals were given free access to water and diet. All sections of study adhered to the ARRIVE Guidelines. All the animal studies (including the rat euthanasia procedures) were conducted according to the UK Animals (Scientific Procedures) Act, 1986 and Directive 2010/63/EU in Europe. The sample size in the results was chosen to be consistently used by others in this research direction. ROT was solubilized in 2% DMSO (1 mg/kg/d) and injected intraperitoneally once daily in rats for 21 consecutive days to establish a PD model. Normal saline with 2% DMSO was used as the control. L-3,4-dihydroxyphenylalanine (L-DOPA)/benserazide was intraperitoneally administered daily (L-DOPA: 6 mg/kg/day+benserazide 15 mg/kg/day, dissolved in saline) for 21 days. Behavioral evaluation of rotarod test was examined on day 1, day 7, day 14, day 21 after initiating L-DOPA/benserazide treatment to confirm the loss of dopamine in ROT-treated rats. In the group receiving ARG treatment, rats were given drinking water containing 2% ARG freely without controlling the amount of water. And the daily water intake of rats remained consistent across the different groups. Sodium nitroprusside (SNP, Sigma‒Aldrich, BP453) as a traditional NO donor, were dissolved in phosphate buffered saline for animal experiments. In the group receiving NO donor administration, 0.1 mg/kg SNP was intraperitoneal injected to rats for 21 consecutive days according to the previous study [20]. A randomized ARG/SNP and vehicle treating schedule was created using the standard = RAND () function in Microsoft-Excel and prepared by a third person not involved in the experiment to maintain blinding. PD model, experiment performing and data analysis were conducted by different persons to keep blinding.

### Double-label immunofluorescence staining

To explore the distribution of DDAH-1 and its co-expression with neurons or astrocytes in the brain, and the colocalization of mitochondria and ER in cells, double-label immunofluorescence staining was performed. Following washing, fixation and blocking, the frozen brain sections of WT rats or slides with cells were obtained and incubated with primary antibodies DDAH-1 (Abcam, ab180599, 1:100), TH (Abcam, ab315207, 1:1000), glial fibrillary acidic protein (GFAP, Abcam, ab279290, 1:50), tubulin beta 3 (TUBB3, Abcam, ab78078, 1:500), translocase of outer mitochondrial membrane 20 homolog (TOMM20, Abcam, ab56783,1:200), Calnexin (Abcam, ab22595, 1:250) and the corresponding secondary antibodies (Jackson Immuno Research,1:500). The sections were viewed, and images were captured at a magnification of ×200 and ×400. The number of DDAH-1+/TH+ cells and DDAH-1+/GFAP+, and the colocalization of TOMM20 and calnexin were analyzed using ImageJ software. Each sample was evaluated in triplicate.

### Measurement of brain ADMA concentrations

Different regions of brain tissue were obtained and weighed. A certain amount of PBS (pH 7.4) was added to homogenize the specimen fully by homogenizer. Centrifuge for about 20 min (2000–3000 rpm) and collect supernatant carefully. Then each sample was measured by ELISA kit (JianglaiBio, Shanghai, China) following the manufacturer’s instructions and evaluated in triplicate. A standard curve of six standard concentrations and their corresponding OD values (450 nm) was generated to determine the concentration of the unknown sample. Each sample was evaluated in triplicate.

### Terminal dUTP nick-end labeling staining

Cell apoptosis was analyzed using fluorometric terminal dUTP nick-end labeling (TUNEL) in situ cell death detection kit (Roche, 11684795910, Germany) following the manufacturer’s instructions. Briefly, 10-μm-thick frozen sections of targeted brain regions were fixed and permeabilized. Each brain slide was added with fluorescein-conjugated TUNEL reaction mixture incubated at 37 °C for 60 min without light. The sections were also mounted with 4′,6-diamidino-2-phenylindole (DAPI) to visualize nuclei. The DAPI and TUNEL positive cells were found to be late apoptotic cells by microscopy. Each sample was evaluated in triplicate.

### Rotarod test

Before ROT injection, animals were pre-trained on the rotating rod for 3 days to enhance the ability to maintain balance and motor coordination. The rats were evaluated again on the next day after the last ROT/DMSO injection. Following adaptation to the horizontal rod, all rats were tested at a start speed of 4 rpm up to a maximum of 40 rpm for a maximum of 5 min. The rotarod tests were performed three times in the morning on day 21 after ROT injection. The latency of the rats to maintain their balance on the rod were measured.

### Open field test

The test was performed in an open-field area. The floor was divided into 25 equal squares to form a 5 × 5 grid pattern. The rat was placed in the chamber for 5 min to acclimatize. Then, the rat’s movement was recorded on the video, and the rest time of the animal was quantified using automatic software. The test was performed for three times on day 21 after ROT injection under dim white light in a sound-attenuated room, and the floor and wall were cleaned with 95% ethyl alcohol after each test. The moving distance, moving speed, ambulation frequency (number of squares crossed with both forepaws) were measured.

### Grip strength

To assess the strength of forelimb, this test is based on the tendency of a rat to instinctively grasp a grid when suspended by the bod. The test apparatus (Grip Strength Meter, Columbus, USA) consisted of a grasping bar attached to a force transducer, which could measure the maximum force of the rat during the pull. The unit of force used is grams of force. Each animal was handled via the body and brought near the bar, allowing the grasping of the grid with both forepaws and then gently pulled back until they released it. Measurements were discarded when rats used its hind paws, turned backwards during the pull, only used one paw, or released the bar without resistance. Animals were trained and tested on two consecutive days, using the same protocol. Five such measurements were obtained for each animal, and the resting period between each pull was 1 min. The three best of each session were averaged and used for analysis. Results were expressed as total force (g).

### Brain tissue protein extraction

The rats were euthanized by overanesthesia and the brain tissue was quickly removed on ice after decapitation. The column tissue&cell protein extraction kit (EpizymeBiotech, PC201plus) was used to extract brain tissue protein. The experimental procedures were roughly as follows. 15–20 mg brain tissue was taken into the purification column, rotated and ground 50–60 times. Two hundred microliters denatured lysate was added. After grinding for 30–50 times and incubating at room temperature for 2 min, the samples were centrifuge in 4 °C for 2 min, rotation speed 14,000 rpm. The supernatant was absorbed finally.

### Immunohistochemistry

To count the number of dopaminergic neurons, TH staining of serial coronal paraffin midbrain and striatum (Str) sections (40-μm thickness) were obtained from each brain using a cryostat (CM1850, Leica, Germany) at −20 °C and collected in PBS. Serial sections were selected to the Nissl or TH staining method. For TH immunohistochemistry, the sections were incubated with TH antibody (Abcam, ab137869, 1:500), biotin-labeled secondary antibody, and DAB solution. The images for TH staining were collected at room temperature with an inverted fluorescence microscope (E400, Nikon) under a 4× objective lens (Nikon) equipped with a digital microscope camera. The total number of TH-positive neurons in every fourth section of the entire SNpc was counted stereologically with the Stereo Investigator software (MBF Bioscience, Williston, VT, USA), based on the fractionator method. Five brains from each group were analyzed for cell counting, and the data presented as the total number of TH-positive neurons in the SNpc of each brain. The Str TH level was quantified by densitometry and sections were scanned in a high-resolution scanner. The Str boundaries were traced and the optical density was measured in terms of grey levels using ImageJ software. For the Nissl staining method, sections were mounted on gelatin-coated slides. Following being rehydrated, they were stained with 0.02% cresyl violet in acetate buffer. The numbers of Nissl-stained neurons were assessed via the cell counter tool of ImageJ software and expressed as the percentage of control rats.

### Determination of dopamine in the Str by HPLC

The Str tissue was weighed and homogenized with ice-cold homogenate including 0.1 M perchloric acid and 0.1 mM EDTA-2Na. After centrifugation at 20,000 r/min at 4 °C for 20 min, the supernatant was collected, filtered through a 0.22 µm Millipore filter and injected into the HPLC-Electrochemical Detector system (Waters e2695/2465, USA) for the measurement of dopamine. The injection volume was 20 μL and the flow rate was set at 1 mL/min. Each sample was evaluated in triplicate.

### Statistical analysis

All data were expressed as mean ± standard deviation (SD) and analyzed using Prism 8 (GraphPad Software). Normality was assessed using the Shapiro-Wilk test. Variances were assessed with the Bartlett test for normally distributed data. For comparisons of two groups with normal distributions and equal variances, two-tailed unpaired *t*-test was used. For the comparison of multiple groups with normal distribution and equal variance, one-way or two-way analysis of variance (ANOVA) with Tukey post-hoc testing was used. n.s. means no significance. *p* < 0.05 was considered statistically significant (*p < 0.05, **p < 0.01, ***p < 0.001).

## Results

### DDAH-1 was highly expressed in SN dopaminergic neurons

**Fig. 1:**
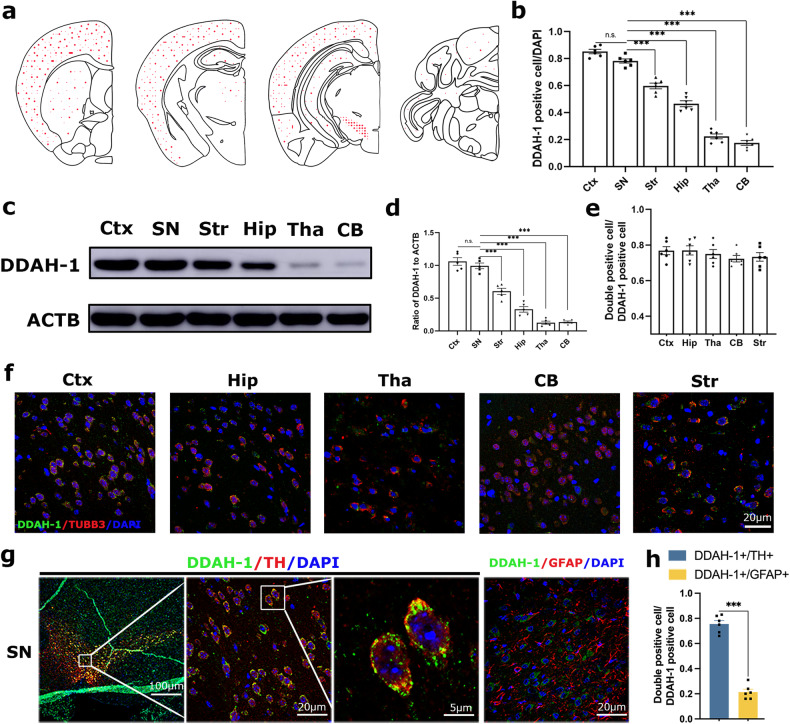
DDAH-1 was highly expressed in dopaminergic neurons of SN. **a** Representative maps showing DDAH-1 expression (red dots) in different brain regions of rats. **b** Quantitative analysis of DDAH-1 immunofluorescence staining (expressed as the ratio of DDAH-1-positive cells to total counted cells) in various brain regions, including the Ctx, SN, Str, Hip, Tha, and CB. F(5, 30) = 228.5, *p* < 0.0001. **c** Immunoblot and **d** semi-quantitative analysis of DDAH-1 expression in Ctx, SN, Str, Hip, Tha and CB. F(5, 24) = 101.4, *p* < 0.0001. **e** Quantitative analysis and **f** representative images of immunofluorescence staining of DDAH-1 and TUBB3 in Ctx, Hip, Tha, CB and Str. The number of DDAH-1+/TUBB3+ cells normalized to the total number of DDAH-1+ cells indicated DDAH-1 was expressed in TUBB3-positive neurons. F(4, 25) = 0.8007, *p* = 0.5362. **g** Representative images and **h** quantitative analysis of DDAH-1/TH and DDAH-1/GFAP co-immunofluorescence staining in the SN in DDAH-1^+/+^ rats. The number of DDAH-1+/TH+ cells or DDAH-1+/GFAP+ normalized to the total number of DDAH-1+ cells indicating that DDAH-1 was mainly expressed in TH+ neurons. Results are presented as mean ± SD, *n* = 5–6 independent experiments. Statistical significance was determined using one-way ANOVA (**b**, **d**, **e**) with Tukey post-hoc testing or unpaired t test with two-tailed *p* value (**h**). Significance levels are indicated as n.s. *p* > 0.05, ***p < 0.001. Ctx cortex, SN substantia nigra, Str striatum, Hip hippocampus, Tha thalamus, CB cerebellum, DDAH-1 dimethylarginine dimethylamino hydrolase-1, ACTB β-actin, DAPI 2-(4-Amidinophenyl)-6-indolecarbamidine dihydrochloride, TUBB3 tubulin beta 3, TH tyrosine hydroxylase, GFAP glial fibrillary acidic protein.

Our previous study has shown DDAH-1 was mainly located in cerebral cortex (Ctx) [8]. Here, more brain regions include Str, SN, hippocampus (Hip), thalamus (Tha) and cerebellum (CB) were detected for the distribution of DDAH-1. Interestingly, SN and Ctx exhibited significantly higher levels of DDAH-1 protein detected by either immunostaining (expressed as the ratio of DDAH-1-positive cells to total counted cells) in Figs. S1b, 1a, b or immunoblot in Fig. 1c, d compared to other brain regions. From the perspective of cellular morphology, DDAH-1 positive cells exhibit neuronal morphology. Using TUBB3, representing for neuronal cytoskeleton to label neurons, we found that DDAH-1 co-expressed with neuronal markers in various brain regions (Fig. 1e, f).

To note, DDAH-1 was strongly co-localized with TH, a marker for dopaminergic neurons, in SN while fewer signals were found in astrocytes (Fig. 1g, h). Since DDAH-1 is the main hydrolytic enzyme for ADMA, concentrations of ADMA were measured in different brain regions afterwards. In DDAH-1^+/+^ rats, relatively lower ADMA concentrations were examined in both Ctx and SN where DDAH-1 expression was rather high (Fig. S1c). When rats were deplted the first exon of DDAH-1, ADMA levels in all regions were significantly higher than those in DDAH-1^+/+^ rats while there was little diversity among different brain regions (Fig. S1c). These findings suggested that DDAH-1 was enriched in dopaminergic neurons in the SN, potentially regulating ADMA levels. Consequently, further exploration into the potential roles of DDAH-1 and ADMA in PD pathology is warranted.

### DDAH-1 depletion aggravated ROT-induced dopaminergic neurodegeneration

**Fig. 2:**
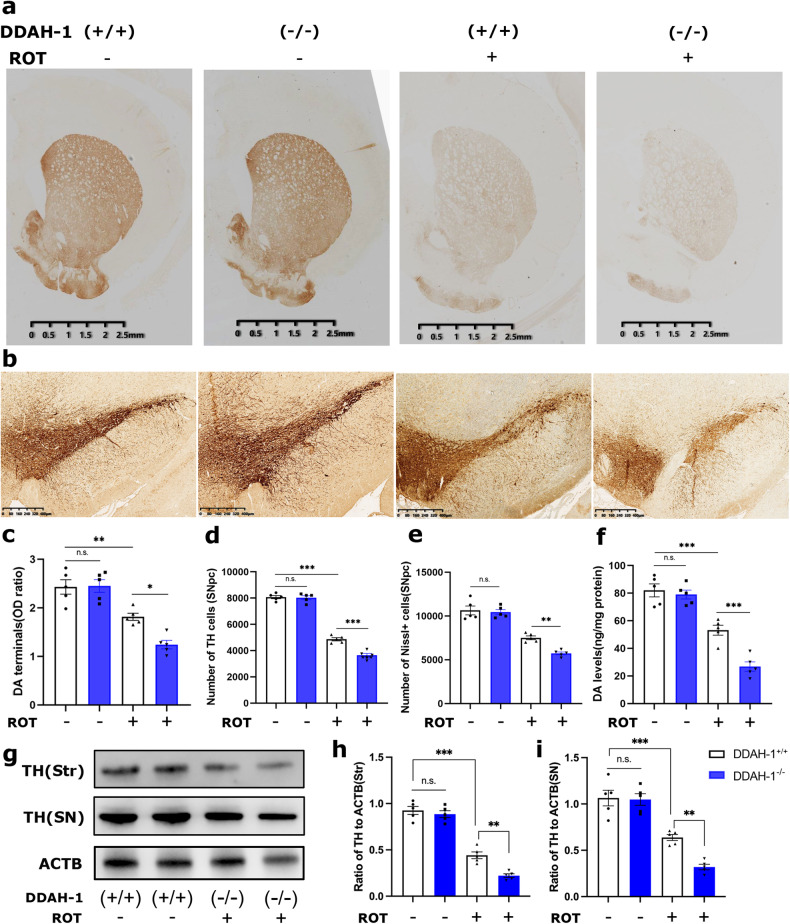
DDAH-1^-/-^ rats showed aggravated dopaminergic neuron degeneration after ROT injection. **a**, **b** Representative images of immunochemical staining for TH-positive neurons in **a** Str and **b** SNpc. **c**, **d** Measurement of striatal dopamine terminals and TH-positive neurons levels, including **c** the optical density (OD) of dopamine terminals, F(3, 16) = 25.15, *p* < 0.0001 and **d** stereological cell counting of TH-positive neurons, F(3, 16) = 276.0, *p* < 0.0001, revealing a significant decrease in DDAH-1^-/-^ rats after ROT injection. **e** The number of Nissl-positive neurons in the SNpc. F(3, 16) = 55.10, *p* < 0.0001. **f** Total Str dopamine levels were measured by HPLC. F(3, 16) = 46.56, *p* < 0.0001. **g** Immunoblot and **h**, **i** semi-quantitative analysis of TH expression in Str and SN. **h** F(3, 16) = 96.06, *p* < 0.0001, **i** F(3, 16) = 39.27, *p* < 0.0001. Results are presented as mean ± SD, *n* = 5 rats/group. Statistical significance was determined using one-way ANOVA with Tukey post-hoc testing. Significance levels are indicated as n.s. *p* > 0.05, *p < 0.05, **p < 0.01, ***p < 0.001. ROT rotenone, DDAH-1 dimethylarginine dimethylamino hydrolase-1, DA dopamine, OD optical density, TH tyrosine hydroxylase, SNpc substantia nigra pars compacta, SN substantia nigra, Str striatum, ACTB β-actin.

To investigate the function of DDAH-1 in PD pathology, we evaluated the histopathological and behavioral differences between DDAH-1^-/-^ and DDAH-1^+/+^ rats in ROT-induced PD model. ROT-injected DDAH-1^+/+^ rats exhibited approximately 50% loss of TH-positive neurons in SN and about 30% reduction of TH-positive terminals in Str compared with DMSO-injected controls (Fig. 2a–d). While DDAH-1^-/-^ rats showed more severe loss of either dopaminergic neurons in SN or the striatal dopaminergic fibers compared to their WT littermates after ROT neurotoxicity (Fig. 2a–d). Nissl cell count indicated that the loss of TH was due to cell loss rather than downregulation of phenotypic markers (Fig. 2e). HPLC analysis showed the striatal dopamine content in DDAH-1^-/-^ rats was significantly reduced compared to DDAH-1^+/+^ rats after ROT injection (Fig. 2f). Alterations of TH protein in homogenate of both SN and Str in all groups were consistent with the results of TH immunostaining (Fig. 2g–i). Behaviorally, ROT-injected DDAH-1^+/+^ rats had a significant decline in both locomotor function (Fig. S2a–e) and muscle strength (Fig. S2f), while DDAH-1^-/-^ rats performed even worse. Alleviation of the motor deficit after L-DOPA/benserazide administration indicated a loss of dopamine in ROT-treated rats (Fig. S2g). No significant damage occurred to other cells, including but not limited to neurons, in crucial brain regions following ROT treatment (Fig. S2h).

**Fig. 3:**
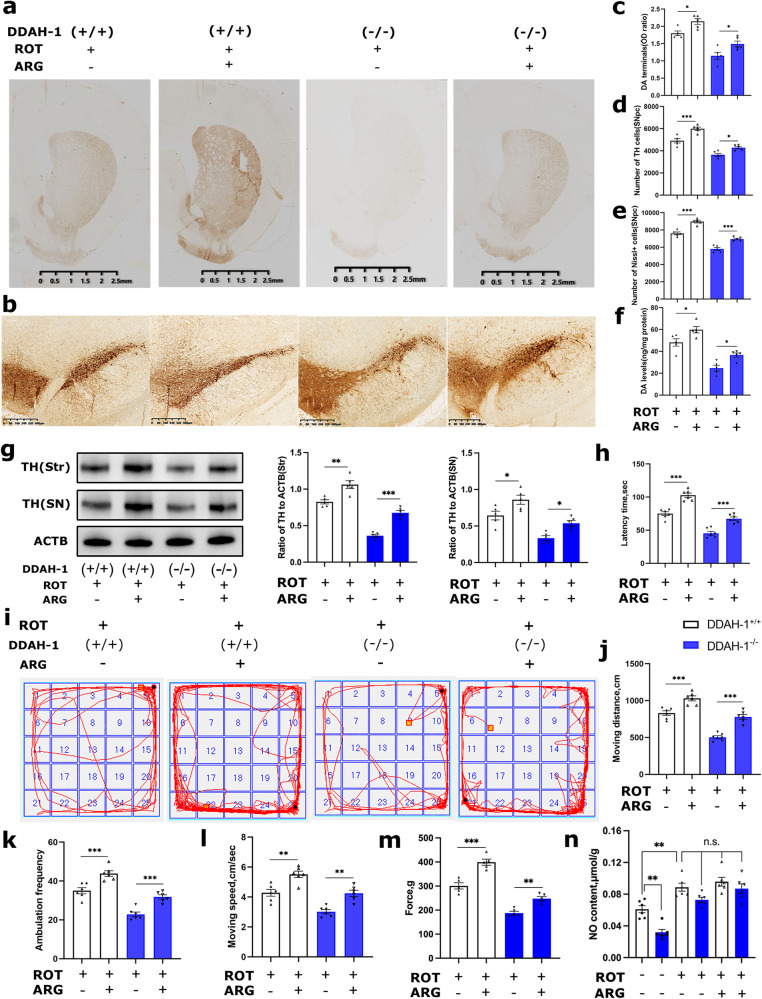
Supplementing ARG to DDAH-1^-/-^ rats alleviated ROT-induced dopaminergic neurodegeneration. **a**, **b** Representative immunochemical staining images for TH-positive neurons in **a** Str and **b** SNpc. **c**, **d** Striatal dopamine terminals and TH-positive neurons levels after ARG treatment, including **c** the OD of dopamine terminals, F(3, 16) = 25.77, *p* < 0.0001 and **d** stereological cell counting of TH-positive neurons, F(3, 16) = 43.91, *p* < 0.0001. **e** The number of Nissl-positive neurons in the SNpc after ARG supplement. F(3, 16) = 79.05, *p* < 0.0001. **f** Total Str dopamine levels were also elevated after ARG supplement. F(3, 16) = 29.83, *p* < 0.0001. **g** Immunoblot and semi-quantitative analysis of TH expression in Str and SN after ARG treatment. (Str) F(3, 16) = 66.22, *p* < 0.0001, (SN) F(3, 16) = 19.26, *p* < 0.0001. **h** Quantitative analysis of rotarod tests. F(3, 20) = 64.82, *p* < 0.0001. **i**–**l** The open field recordings (**i**) and quantitative analysis of moving distance (**j**) F(3, 20) = 52.43, *p* < 0.0001, ambulation frenquency (**k**) F(3, 20) = 42.06, *p* < 0.0001 and moving speed (**l**) F(3, 20) = 26.38, *p* < 0.0001. **m** Quantitative analysis of grip strength. F(3, 20) = 76.03, *p* < 0.0001. **n** Quantitative analysis of NO concentrations in midbrain before and after ROT or ARG treatment. F(5, 30) = 24.53, *p* < 0.0001. Results are presented as mean ± SD, *n* = 5–6 rats/group. Statistical significance was determined using one-way ANOVA with Tukey post-hoc testing. Significance levels are indicated as n.s. *p* > 0.05, *p < 0.05, **p < 0.01, ***p < 0.001. ROT rotenone, ARG l-arginine, DDAH-1 dimethylarginine dimethylamino hydrolase-1, DA dopamine, OD optical density, TH tyrosine hydroxylase, SNpc substantia nigra pars compacta, SN substantia nigra, Str striatum, ACTB β-actin, NO nitric oxide.

DDAH-1 depletion resulted in elevated ADMA activity to inhibit ARG/NO pathway. To explore the relationship of neurodegeneration in DDAH-1^-/-^ PD rats with NO, ARG was supplemented while ROT modeling. Both histopathological (Fig. S3a–d**)** and behavioral tests (Fig. S3e–i**)** confirmed no effects of ARG intake on both DDAH-1^-/-^ and DDAH-1^+/+^ rats without ROT treatment. Remarkably, the administration of ARG significantly attenuated striatal TH^+^ fibers damage (Fig. 3a, c, g) and dopaminergic cell loss (Fig. 3b, d, g) in ROT-injected rats, but the improvement in DDAH-1^-/-^ rats had not yet recovered to the level equivalent to DDAH-1^+/+^ rats (Fig. 3a–g). Similar protection of ARG against ROT exposure was observed in motor behavior (Fig. 3i–l) and muscle strength (Fig. 3m) of DDAH-1^-/-^ rats. NO concentrations were then examined to assess the role of ARG/NO pathway. Data showed NO levels in the midbrain were lower in DDAH-1^-/-^ than WT rats (Fig. 3n). After ROT injection, NO levels were elevated while there was no significant difference between DDAH-1^-/-^ rats and DDAH-1^+/+^ rats (Fig. 3n). Given ARG treatment did not lead to a significant increase in NO levels in vivo (Fig. 3n), we employed SNP, a traditional NO donor that directly supplies NO without relying on its synthesis pathway. Despite more NO provision, SNP-injected rats presented similar behavioral and pathological outcomes with ARG-treated groups in both PD and control rats (Figs. S3–4).

**Fig. 4:**
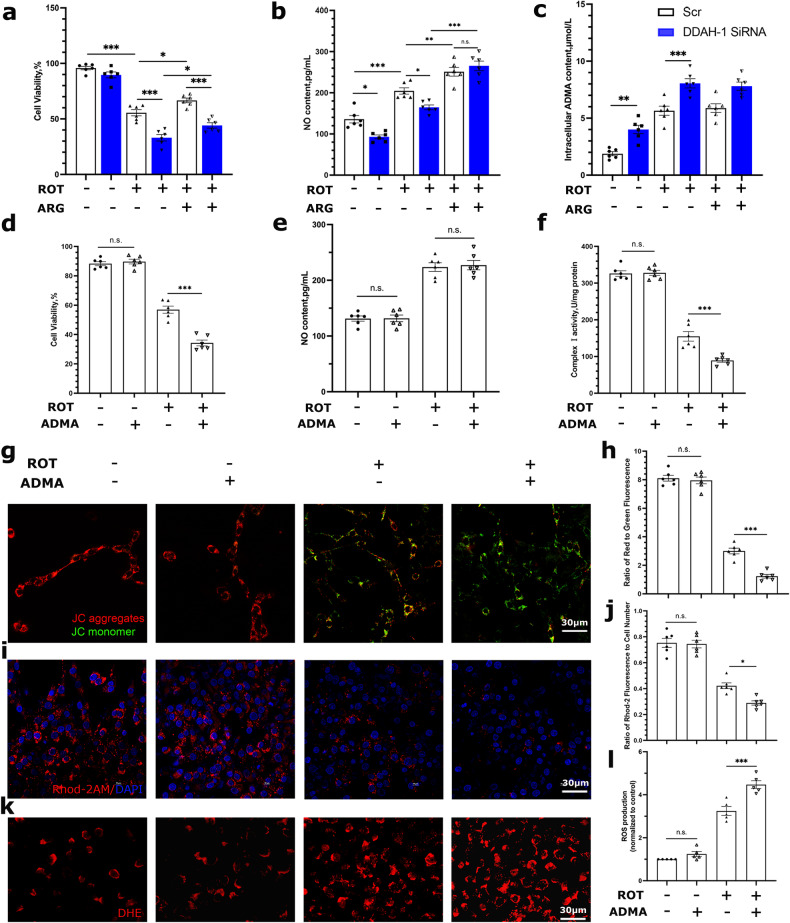
DDAH-1 knockdown exacerbated ROT-induced mitochondrial dysfunction via increasing ADMA. **a**–**c** Quantification of the **a** cell viability, F(5, 30) = 103.7, *p* < 0.0001, **b** NO levels, F(5, 30) = 61.87, *p* < 0.0001 and **c** intracellular ADMA concentrations, F(5, 30) = 43.12, *p* < 0.0001. **d**–**f** Quantifications of **d** cell viability, F(3, 20) = 199.3, *p* < 0.0001, **e** NO content, F(3, 20) = 62.52, *p* < 0.0001 and **f** activity of MRCC I, F(3, 20) = 195.9, *p* < 0.0001. **g**, **h** Representative images and quantitative analysis of mitochondrial membrane potential (MMP) measurement by JC-1 staining. Red aggregates indicate normal MMP while green monomers indicate disrupted MMP. F(3, 20) = 316.4, *p* < 0.0001. **i**, **j** Representative images of mitochondrial Ca^2+^ displayed by red Rhod-2AM staining and its quantitative analysis. Rhod-2 fluorescence normalized by DAPI-positive cells. F(3, 20) = 77.59, *p* < 0.0001. **k**, **l** Representative images of DHE staining for ROS measurement and statistical analysis of ROS fluorescence intensity normalized to the control. F(3, 16) = 123.8, *p* < 0.0001. Results are presented as mean ± SD, *n* = 5–6 independent experiments. Statistical significance was determined using one-way ANOVA with Tukey post-hoc testing. Significance levels are indicated as n.s. *p* > 0.05, *p < 0.05, **p < 0.01, ***p < 0.001. ROT rotenone, ARG l-arginine, NO nitric oxide, ADMA asymmetric dimethylarginine, DDAH-1 dimethylarginine dimethylamino hydrolase-1, siRNA small interfering RNA, Scr scramble, ROS reactive oxygen species, DHE dihydroethidium.

Collectively, these results suggested that DDAH-1 depletion exacerbated ROT-induced neurodegeneration via abnormal ADMA metabolism, which could be rescured by NO signaling. However, the recovery of DDAH-1^-/-^ rats did not match the performance in DDAH-1^+/+^ rats, despite similar levels of NO in both groups. Therefore, we speculated that there might be other potential DDAH-1/ADMA downstream mechanisms related to neurodegeneration.

### DDAH-1 protected against ROT-induced mitochondrial damage and cell death via ADMA metabolism

In this part, we investigated regulatory effects and potential mechanisms of DDAH-1 in vitro. Treatment with proportional series concentrations of ROT for 24 h resulted in a concentration-dependent cytotoxicity to PC12 cells, as evidenced by Fig. S5a, b. Both RT-qPCR and western blot data confirmed downregulation of DDAH-1 expression on PC12 cells, which had no effects on DDAH-2 expression (Fig. S5c, d).

Consistent with the in vivo data, DDAH-1 knockdown aggravated ROT-induced cell death (Fig. 4a) with deceased NO levels (Fig. 4b) and increased ADMA levels (Fig. 4c). ARG supplementation was able to fully reversed the reduction in NO production resulting from DDAH-1 knockdown (Fig. 4b). However, poorer cell viability by DDAH-1 knockdown was still exhibited compared with the control group (Fig. 4a). Mounting evidence indicates that mitochondrial abnormalities, featured by respiratory chain dysfunction and calcium homeostasis impairment, play a fundamental role in the neurodegerative process of PD [21]. Our data showed that DDAH-1 downregulation exacerbated the ROT-induced reductions of MMP (Fig. S5e, f), MRCC-I activity (Fig. S5g), and mitochondrial Ca^2+^ levels (Fig. S5h, i), and increase of ROS production (Fig. S5j, k). While ARG supplement had no reversal effects on the mitochondrial dysfunction (Fig. S5e–k). These findings suggested that mitochondrial dysfunction was one of the significant neurodegeneration mechanisms triggered by DDAH-1 downregulation, potentially operating independently of NO pathway.

To elucidate the effects of DDAH-1/ADMA on mitochondrial function in ROT-treated cells, the cells were then administered with ADMA which would result in a similar intracellular ADMA level as DDAH-1 knockdown (Fig. S5l). ADMA interference alone did not affect NO production (Fig. 4e). While it exacerbated ROT-induced neuronal death (Fig. 4d), declined MRCC-I activity (Fig. 4f), MMP (Fig. 4h), and mitochondrial Ca^2+^ levels (Fig. 4j), and ROS generation (Fig. 4l), implying that abnormally increased ADMA may mediated ROT-related mitochondrial damage.

**Fig. 5:**
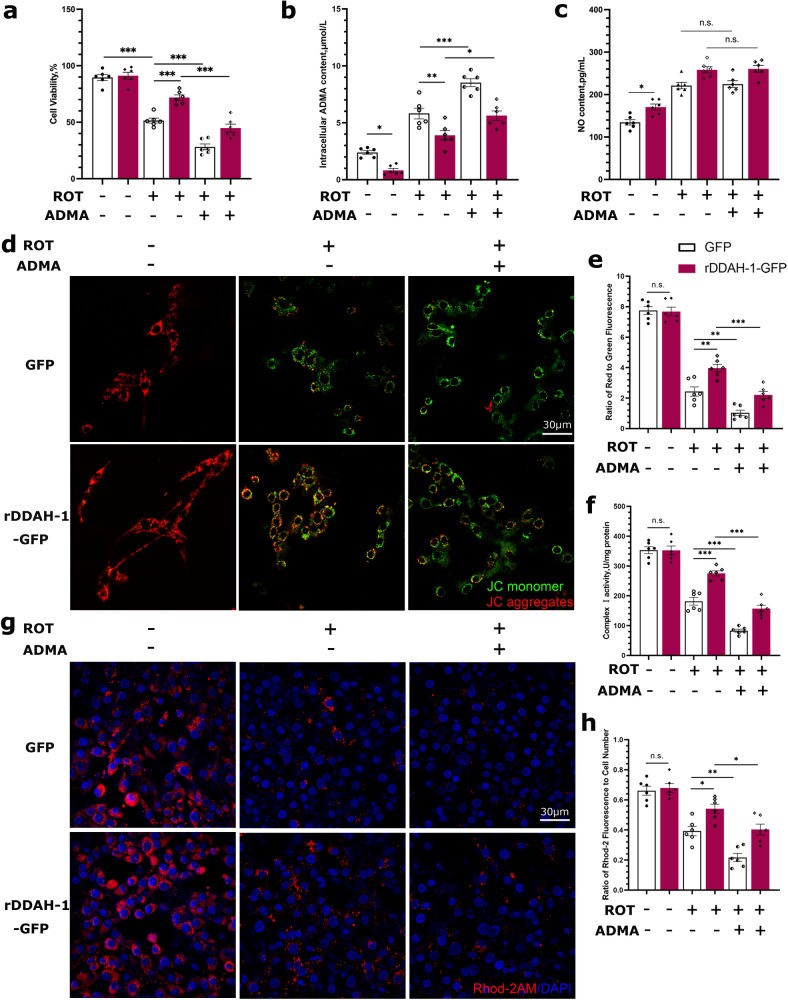
ADMA weakened the protective effect of DDAH-1 overexpression on mitochondrial function. **a**–**c** Quantification of the **a** cell viability, F(5, 30) = 86.82, *p* < 0.0001, **b** ADMA concentrations, F(5, 30) = 61.38, *p* < 0.0001 and **c** NO concentrations, F(5, 30) = 46.11, *p* < 0.0001 showed the alleviated cytotoxicity of ROT by DDAH-1 overexpression with lowered ADMA concentration. ADMA treatment significantly attenuated the protective effects of DDAH-1 overexpression without influencing NO levels. **d**, **e** Representative images of JC aggregates and monomers staining and MMP quantification, which revealed significant attenuation of DDAH-1’s protective effects by ADMA treatment. F(5, 30) = 130.7, *p* < 0.0001. **f** Quantification of MRCC I activity. F(5, 30) = 98.79, *p* < 0.0001. **g**, **h** Representative images and quantitation of mitochondrial Ca^2+^ indicated significant weakening of protection from DDAH-1 overexpression on sustaining mitochondrial Ca^2+^. F(5, 30) = 33.71, *p* < 0.0001. Results are presented as mean ± SD, *n* = 6 independent experiments. Statistical significance was determined using one-way ANOVA with Tukey post-hoc testing. Significance levels are indicated as n.s. *p* > 0.05, *p < 0.05, **p < 0.01, ***p < 0.001. ROT rotenone, ADMA asymmetric dimethylarginine, DDAH-1 dimethylarginine dimethylamino hydrolase-1, GFP green-fluorescent protein.

We further overexpressed DDAH-1 plasmids in ROT-treated PC12 cells followed by ADMA treatment. DDAH-1 overexpression was confirmed via its mRNA and protein levels (Fig. S6a, b). DDAH-2 levels were not changed in DDAH-1 overexpressed PC12 cells (Fig. S6c). DDAH-1 overexpression significantly attenuated ROT-induced cytotoxicity (Fig. 5a), decreased cellular ADMA concentration (Fig. 5b) and increased NO levels (Fig. 5c). Additionally, DDAH-1 upregulation restored the MMP (Fig. 5d, e), MRCC-I activity (Fig. 5f) and mitochondrial Ca^2+^ level (Fig. 5g, h) disrupted by ROT. DDAH-1 upregulation would reduce ROS production (Fig. S6c). However, these beneficial effects of DDAH-1 overexpression on mitochondrial functions were totally abolished by ADMA treatment (Figs. 5d–h, S6c). ADMA treatment had not yet influenced intracellular NO levels (Fig. 5c).

The above results indicated that the neuroprotective effects of DDAH-1 may be associated with its protection against mitochondrial dysfunction via regulation of ADMA levels.

### DDAH-1 via ADMA maintained MAM formation and MFN2 oligomerization to alleviate ROT-induced mitochondrial dysfunction

**Fig. 6:**
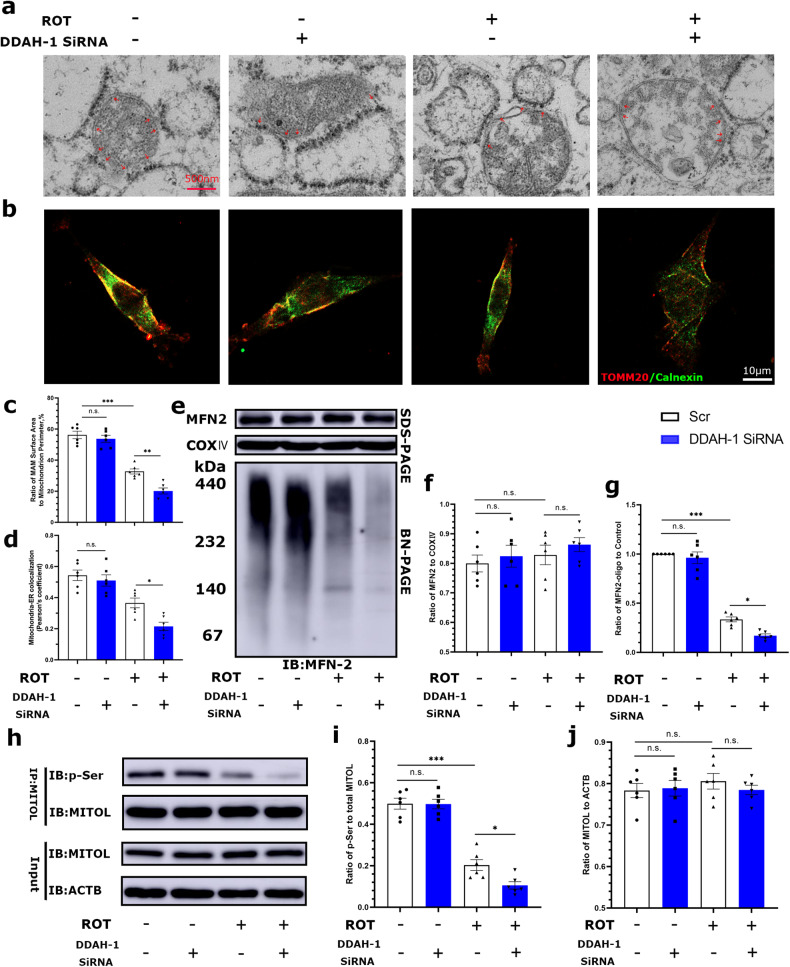
DDAH-1 knockdown exacerbated ROT-induced disruptions of ER-mitochondrial contacts and MFN2 oligomerization. **a** Representative TEM images of the morphology of ER-mitochondrial contacts. Red arrows indicated the ER-mitochondrial surface area. **b** Representative images of co-immunofluorescence staining for TOMM20 and Calnexin. **c** Quantitation of ER length adjacent to mitochondria normalized by mitochondrial perimeter. F(3, 20) = 69.76, *p* < 0.0001. **d** Quantitation of mitochondrial-ER colocalization. F(3, 20) = 21.80, *p* < 0.0001. **e**–**g** The steady-state levels and oligomerization of MFN2 were determined in isolated mitochondria from PC12 cells using BN-PAGE and immunoblotting. Treatment of ROT or DDAH-1 knockdown did not change the steady-state levels of MFN2, while knockdown of DDAH-1 aggravated the reduction of MFN2 oligomerization induced by ROT. **f** F(3, 20) = 0.7133, *p* = 0.5555, **g** F(3, 20) = 162.6, *p* < 0.0001. **h** Total cell lysates were immunoprecipitated with anti-MITOL and immunoblotted with p-Ser antibodies. Representative immunoprecipitation/immunoblot of phosphorylated MITOL. **i**, **j** Quantitive data showed that ROT treatment decreased the phosphorylation of MITOL at serine, which was further reduced following DDAH-1 knockdown. **i** F(3, 20) = 73.38, *p* < 0.0001, **j** F(3, 20) = 0.3714, *p* = 0.7745. Results are presented as mean ± SD, *n* = 6 independent experiments. Statistical significance was determined using one-way ANOVA with Tukey post-hoc testing. Significance levels are indicated as n.s. *p* > 0.05, *p < 0.05, **p < 0.01, ***p < 0.001. ROT rotenone, DDAH-1 dimethylarginine dimethylamino hydrolase-1, TOMM20 translocase of outer mitochondrial membrane 20 homolog, MFN2 mitofusin2, COX IV cytochrome c oxidase IV, siRNA small interfering RNA, Scr scramble, MAM mitochondria-associated endoplasmic reticulum membrane, ER endoplasmic reticulum, ACTB β-actin, MITOL mitochondrial ubiquitin ligase.

MAM facilitates Ca^2+^ exchange between ER and mitochondria, playing a crucial role in maintaining mitochondrial homeostasis. Here, we investigated the impact of DDAH-1 on ER-mitochondrial contacts in ROT-treated PC12 cells. TEM analysis showed that ROT alone significantly decreased the coverage of MAM characterized by the length of the mitochondrial surface being in close contact to ER (within a 30-nm distance), whereas DDAH-1 downregulation further reduced the proportion of MAM near the ER surface (Fig. 6a, c). The colocalization of TOMM20 (mitochondrial marker) and calnexin (ER marker) was also analyzed to assess the mitochondrial-ER contact, which exhibited decreasing TOMM20-calnexin overlap after DDAH-1 knockdown (Fig. 6b, d). Conversely, the overexpressed DDAH-1 alleviated this effect, indicating its essential role in preserving MAM formation (Fig. S7a–d).

MFN2 is a key player in establishing efficient MAM formation by oligomerizing into a high-molecular-weight complex that bridges mitochondria to ER [22–24]. To explore the molecular mechanisms behind MAM formation, we analyzed the expression of steady-state MFN2 and its oligomers. Neither ROT nor DDAH-1 knockdown alone altered the steady-state levels of MFN2 (Fig. 6e, f), but MFN2 oligomerization, as detectecd using the same mitochondrial extracts, declined in ROT-treated cells (Fig. 6e, g). This decline was exacerbated by DDAH-1 downregulation (Fig. 6g). Consistent with these data, MFN2 oligomerization was re-established in ROT-treated cells after DDAH-1 overexpression (Fig. S7e–g), suggesting DDAH-1 may prevent MFN2 oligomerization impairment-mediated MAM loss to preserve mitochondrial function via ADMA.

### DDAH-1 via ADMA regulated MAM formation and MFN2 oligomerization probably through MITOL phosphorylation

To further explore the mechanism on MFN2 oligomerization, we focused on MITOL, a mitochondrial ubiquitin ligase that regulates MAM formation by activating MFN2 and maintaining its oligomerization [14]. First, we analyzed total cell lysates using immunoblotting and found no significant difference in the expression of total MITOL after ROT treatment or DDAH-1 knockdown (Fig. 6h, j, Input panel). When total cell lysates were immunoprecipitated with anti-MITOL and immunoblotted with p-Ser antibodies, ROT treatment decreased the phosphorylation of MITOL at serine, which was further reduced following DDAH-1 knockdown (Fig. 6h, i). Whereas the overexpression of DDAH-1 mitigated this effect (Fig. S7h–j). The above data suggested a possible role of MITOL phosphorylation in ADMA-mediated MFN2 oligomerization and MAM formation.

## Discussion

PD is characterized by selective neuronal loss in the SNpc, where in-depth mechanisms remain to be identified [25]. Impairment of ubiquitin-proteasome and auto-phago-lysosome pathways, oxidative stress response and mitochondrial dysfunction have been implicated in neuronal dysfunction in PD pathogenesis [25]. Researchers have been motivated to establish specific metabolic biomarkers related to these pathways, which will be helpful in disease monitoring and therapeutic guidance.

ADMA is such a candidate metabolite whose concentration in either serum or cerebrospinal fluid (CSF) differentiates significantly between controls and PD patients. Kirbas et al. found a higher serum ADMA level in newly diagnosed PD patients compared to healthy controls [26]. Despite no similarly comparative inclination about CSF ADMA level detected in Sankowski’s research, patients with motor fluctuations had a more elevated ADMA level in CSF than that of other PD patients. These studies indicated the role of ADMA in the development of PD [27].

Breakdown of the majority of ADMA occurs via DDAH which exists as two isoforms in the cytoplasm [5]. DDAH-1 is most highly expressed in the brain and is co-expressed with neural NOS (nNOS), regarded as the primary isoform responsible for metabolizing ADMA. Instead, DDAH-2 is primarily found in the heart, kidney and placenta, and sparsely distributed in the brain compared to the widely presented DDAH-1. There was an intense signal of DDAH-1 protein observed in the Str, Ctx and Hip of the mouse while a relatively moderate signal was distributed in the Tha and CB [28]. DDAH-1 mRNA in the adult mouse brain distributed primarily in Tha, hypothalamus and pallidum, available at Allen Mouse Brain Atlas and Mouse Brain Atlas from Linnarsson Lab [29, 30]. The discrepancy may be explained by post-transcriptional and post-translational regulation, the differences in stability between mRNA and protein, and the sensitivity of antibodies. Similarly, regional expression of DDAH-1 protein in the human brain was more limited to the Ctx, Hip, basal ganglia and CB compared with the database mRNA expression profile, shown among Allen Human Brain Atlas and the Human Protein Atlas [31, 32]. In our study, we also found a wide distribution of DDAH-1 within the brain among which semi-quantitative analysis illustrated that a higher DDAH-1 expression in either the homogenate or the slices of Ctx than that in Str, Hip, Tha and CB. While DDAH-1 expression in SN was equivalent to that in Ctx, which few studies had reported. It was matched with the relatively lower levels of ADMA in both Ctx and SN. These results indicated a possible relationship between DDAH-1/ADMA and pathomechanism of dopaminergic neurons.

Through co-labeling analysis of DDAH-1 with specific cell markers, DDAH-1 was observed in neurons including multiple subpopulations, especially in the Ctx and Hip of both adult mouse and human brain [29, 31]. Kozlova et al. also found an overlapping signal between DDAH-1 and neuronal marker NeuN in some brain nuclei such as reticular nucleus. DDAH-1 signals were co-localized with both astrocytes and endothelium in hippocampal formation as well [28]. Our previous study confirmed that DDAH-1 was distributed in neurons and endothelial cells in the Ctx [8]. Due to the important distribution of DDAH-1 in SN, we focused on its specific cell type expression in this region. It was discovered that DDAH-1 was strongly co-localized with neurons, particularly with dopaminergic neurons in SN, where fewer signals of DDAH-1 overlapped with astrocytes. These results jointly suggested a possible relationship between DDAH-1/ADMA and pathomechanism of dopaminergic neurons.

Next we depleted the first-exon of DDAH-1 in ROT-induced PD rats and WT littermates, in accompany with increased ADMA levels. Neurotoxins, such as 6-OHDA (6-hydroxydopamine) and MPTP (1-methyl-4-phenyl-1,2,3,6-tetrahydropyridine) were the most used toxic compounds to model PD, which inhibit the complex I of the electron transport chain but have some limitations. ROT, was selected in this study since it elicits relatively moderate progress of dopaminergic neurodegeneration compared with the other two neurotoxins, and induces α-synuclein aggregates lacking in most toxin-based models [33, 34]. The DDAH-1 deletion in WT rats did not influence on locomotor activity and muscle strength, consistent with that in DDAH-1 KO mouse constructed by a fourth-exon deletion. These KO mice exhibited unaltered general locomotor activity while reduced sensitivity to amphetamine indicating impaired dopamine system [35]. In our study, DDAH-1 deletion exacerbated impaired locomotor function and dopaminergic neurodegeneration in PD rats. It did not additionally induce other cellular damages under a relatively modest concentration of ROT. Moreover, Kozlova et al. suggested DDAH-1 influenced animal behavior and the dopamine system, most probably via the NO pathway [35]. However, the study lacked experimental evidence for the impact of DDAH-1 loss on the brain or specific region changes in NO levels. We found that NO levels in the midbrain were higher in ROT-injected rats than that in WT littermates, and were lower in DDAH-1^-/-^ than WT rats. However, DDAH-1 deletion did not significantly reduce NO levels in ROT-injected rats. It suggested that the NO pathways may mediate damage in neurons exposed to ROT while the mechanisms of neurodegeneration caused by DDAH-1 loss could be more complex including but not limited to the NO pathways.

Then, we supplemented ARG, the precursor for NO formation, for all groups and observed improved outcomes in ROT-injected rats regardless of DDAH-1 KO. Notwithstanding ARG treatment had not yet dramatically elevated NO levels, this departure from general ARG/NO metabolism may be due to the NO examination being limited to the midbrain and potentially other metabolic pathways of ARG. To exclude these, SNP, as a traditional NO donor were employed, which directly provide NO. SNP-injected PD rats presented similar behavioral and pathological outcomes with ARG-treated groups. NO expression from either ARG synthesis or SNP provision seemed to be helpful for damage recovery, but it could recover neither histopathological nor behavioral results of DDAH-1^-/-^ rats to the level of DDAH-1^+/+^ rats, despite almost the same levels of NO in these two groups. In a nutshell, results in vivo indicated that NO may play in a dual roles in PD and inhibition of its synthesis may be an essential part of the mechanisms behind the deterioration of ROT-induced neurodegeneration in DDAH-1 deletion animals.

To figure out the roles of ADMA metabolism and NO synthesis in ROT-induced neurodegeneration, we regulated DDAH-1 expression in PC12 cells. Neither DDAH-1 knockdown nor overexpression showed significant effect on DDAH-2 expression, indicating DDAH-2 might not influence the NO concentration in our study. DDAH-1 depletion increased ADMA levels, decreased NO generation and exacerbated neural damage while DDAH-1 overexpression was in reverse. The supplement of ARG was found to fully restore NO levels and to provide certain neural protection against ROT toxicity. It corresponded with the results from ARG-injected PD rats, implying that a certain level of NO is essential for neural survival. Despite the facts that peroxynitrite (ONOO-), a powerful oxidant derived from NO, will lead to the deterioration of dopaminergic neurons, moderate amounts of NO expression is also proposed to be important for neuronal survival, regeneration, and plasticity [36, 37]. As reported in both our study and Nakagomi’s, the production of NO in damaged neurons may be insufficient to cause any further pathological impairment, but would be a part of the cells’ efforts to survive [38].

It indeed remains controversial that ADMA and NO levels in different conditions, which may be owing to many influencing factors such as varied specimen sources and disease stages. Researchers have reported that ADMA was increased in plasma of the patients with Alzheimer’s disease (AD), accompanied by a highly significant decrease in the plasma concentration of NO [39]. However, there were decreasing ADMA levels in CSF in both AD and amyotrophic lateral sclerosis patients, but a lack of NO levels measurement was in these studies [40, 41]. Researchers explained that elevated ADMA in plasma may be a contributing factor for AD through inhibition of NO production in endothelial cells. Instead, declined ADMA may mediate neural damage by a cerebral increase of NO to form ONOO-. However, serum levels of both ADMA and NO in PD patients were significantly higher than those in healthy controls [26]. Researchers attempted to explain increased NO level for a reactive increase in DDAH-1 levels or enzyme activity resulting from increased levels of ADMA in the brain. Despite no further investigation of DDAH-1 levels in PD, up-regulated DDAH-1 has been observed in the caudate of patients with Huntington’s disease [42].

As similar to results in vivo, increased levels of NO could not rescue DDAH-1-downregulated cells to the normal viability and not prevent their mitochondrial dysfunctions. However, it may be different from the results that ARG could significantly rescue mitochondrial respiration inhibited by ADMA treatment in endothelial cells [15]. Xiong et al. also investigated that ADMA treatment suppressed mitochondrial function likely via the disorder of DDAH-2/endothelial NOS/NO pathway in cardiomyocytes [43]. It may be explained by the timing effect which means either cell viability or mitochondrial function needs more time to be fixed despite of normalized NO levels after the ARG supplement. Meanwhile, there may exist NO-independent pathways which exert synergistic effects with NO-dependent pathways. We confirmed that ADMA treatment with wild-type cells could induce similar cell death and mitochondrial dysfunctions seen in DDAH-1 depleted cells, and could significantly worsen the decreased cytotoxicity and recovered mitochondrial functions in DDAH-1 overexpressed cells. Besides NO pathways, DDAH-1/ADMA metabolism may influence neural survival through direct regulation of mitochondrial functions.

Mitochondrial dysfunction, a well-established mechanism in the development of PD, has been found to be in association with alterations of ER-mitochondria signaling [44, 45]. It has pleiotropic effects on intracellular pathways resulting in respiratory chain damage, calcium disbalance, synaptic transmission reduction, and other consequences present in PD [46–48]. Thus, we further investigated the effect of DDAH-1/ADMA in ER-mitochondria contacts. And it was identified that reduced ER-mitochondrial contacts of ROT-treated cells could be aggravated by DDAH-1 knockdown while be rescued by DDAH-1 overexpression. MFN2 is a crucial protein involved in maintaining the mitochondrial network and mediating the tethering of ER and mitochondria [22, 49]. Loss of MFN2 impaired the formation of MAMs and reduced cells’ ability to survive against energy deficiency induced by 12 h or more durations of ROT treatment [50]. However, there has been a controversy regarding whether MFN2 expression promotes or inhibits ER-mitochondrial tethering [23, 51–53]. MFN2 oligomerization is widely accepted to mediate MAM formation [54, 55]. Our study showed that DDAH-1/ADMA axis regulated MAM dynamics by altering MFN2 oligomerization instead of its expressions.

Basso et al. discovered that ubiquitination controlled the oligomerization of MFN2 and its association with the ER [24]. MITOL, a mitochondrial membrane-associated ubiquitin ligase, could react with MFN2 and lead to its ubiquitination, enhancing ER-mitochondrial contacts [56–58]. Therefore, it was hypothesized that the DDAH-1/ADMA axis could regulate the oligomerization of MFN2 and formation of MAM through MITOL. Toyofuku et al. found that the activity of E3 ubiquitin ligases toward MFN2 was increased by phosphorylation [59]. In this study, serine phosphorylation of MITOL was largely decreased after ROT treatment, accompanied by compromised MFN2 ubiquitination, downregulated MFN2 oligomerization, and failed MAM formation. The decrease in serine phosphorylation of MITOL was aggravated in DDAH-1-downregulated cells while mitigated by DDAH-1 overexpression. These results suggested that the DDAH-1/ADMA axis may be involved in MITOL phosphorylation to regulate MFN2 oligomerization, MAM formation, and mitochondrial function.

## Conclusions

Our research has revealed that DDAH-1 was situated in TH-positive neurons in SN, indicating its involvement in regulating PD pathology. Our innovative hypothesis proposed that DDAH-1 protected dopaminergic neurons from mitochondrial dysfunction and cell death by decreasing intracellular levels of ADMA levels. Our findings indicated that the neuroprotective impact of DDAH-1 might dependent on its ability to modulate the intracellular ADMA concentration. Although the regulation of NO metabolism by DDAH-1 can partically explain its protective effects, its role in regulation of mitochondrial function might be independent of the NO pathway. This process includeed increasing MITOL phosphorylation, which supported MFN2 oligomerization and ER-mitochondrial contacts. By highlighting the correlation between DDAH-1/ADMA and PD, our study provides new insights into the specific loss of SN dopaminergic neurons in PD pathogenesis.

### Supplementary information

Supplemental figures

Full and uncropped western blots

## Data Availability

The datasets used and/or analyzed during the current study are available from the corresponding author on reasonable request.
