# Supplementary material for: DDAH-1 maintains endoplasmic reticulum-mitochondria contacts and protects dopaminergic neurons in Parkinson’s disease
Source: Cell Death Dis. 2024 Jun 7;15(6):399. doi: 10.1038/s41419-024-06772-w (PMC11161642; doi:10.1038/s41419-024-06772-w)
Supplement: Supplementary file 1 — Supplemental figures [file 41419_2024_6772_MOESM1_ESM.pdf]

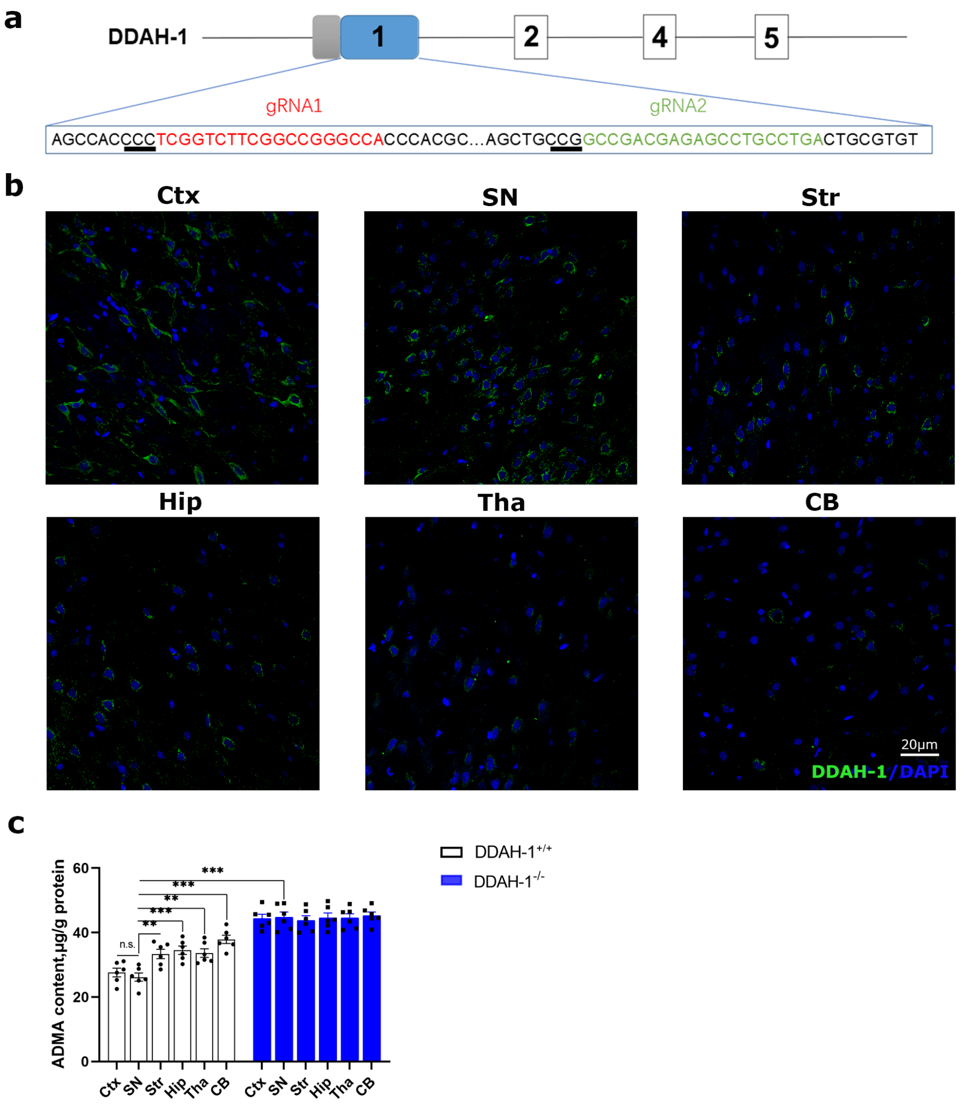

Fig. S1

Generation of the DDAH-1$^{-/-}$ rats and DDAH-1 expression/activity in different brain regions. **(a)** Two guiding RNA (gRNA) chains were designed and constructed in the Exon 1 of DDAH-1. **(b)** Representative images of immunofluorescence staining of DDAH-1 in Ctx, SN, Str, Hip, Tha and CB. **(c)** Quantitative analysis of ADMA concentrations in different brain regions of DDAH-1$^{+/+}$ and DDAH-1$^{-/-}$ rats. Interaction: $F(5, 60) = 5.115$, $p = 0.0006$, row factor: $F(5, 60) = 5.727$, $p = 0.0002$, column factor: $F(1, 60) = 248.9$, $p < 0.0001$. Results are presented as mean ± SD, n=6 rats/group. Statistical significance was determined using two-way ANOVA with Tukey post-hoc testing. Significance levels are indicated as n.s. $p > 0.05$, ** $p < 0.01$, *** $p < 0.001$. Abbreviations: gRNA=guiding RNA, Ctx=cortex, SN=substantia nigra, Str=striatum, Hip=hippocampus, Tha=thalamus, CB=cerebellum, DDAH-1=dimethylarginine dimethylamino hydrolase-1, DAPI=2-(4-Amidinophenyl)-6-indolecarbamidine dihydrochloride, ADMA=asymmetric dimethylarginine.

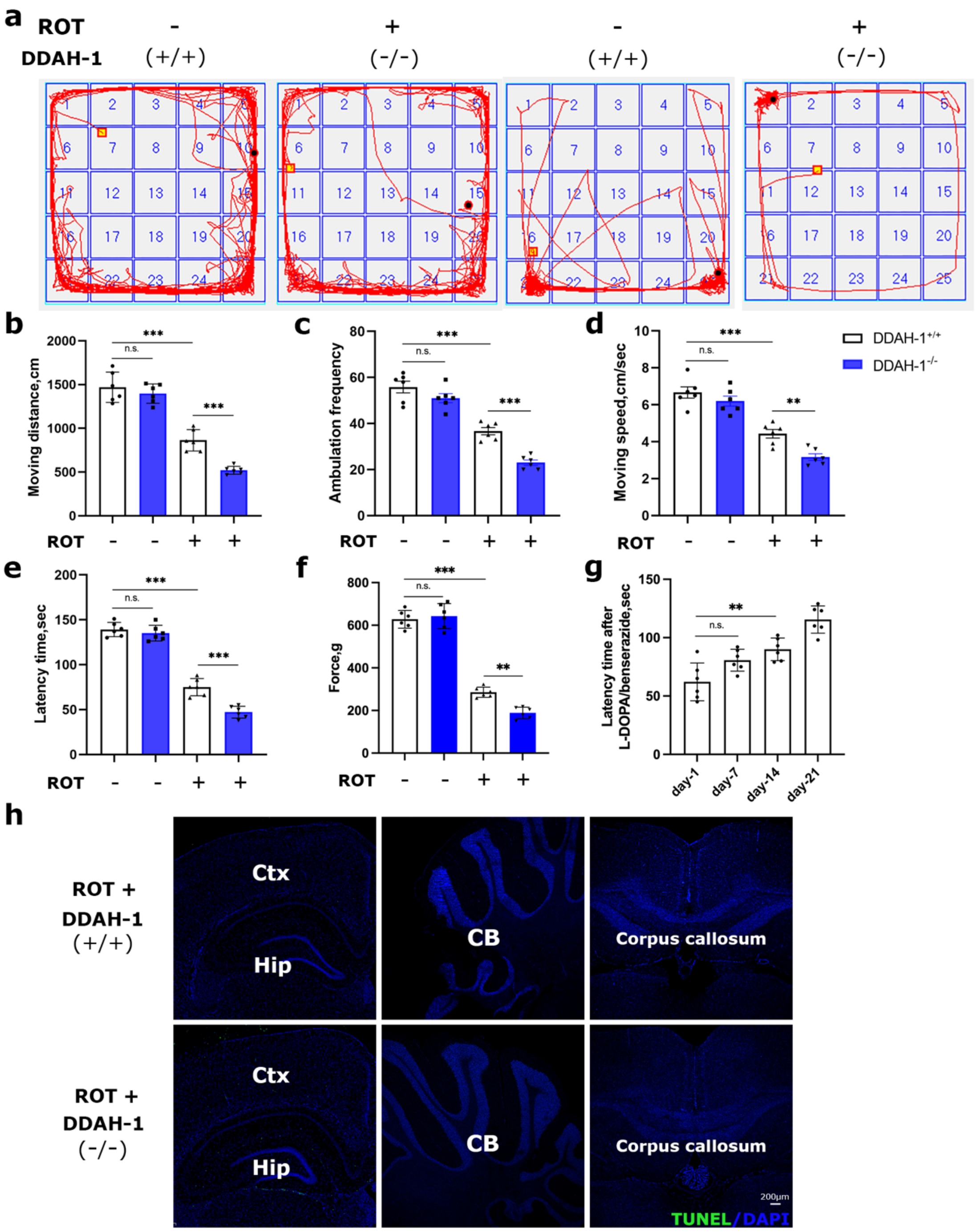

Fig. S2

DDAH-1$^{-/-}$ rats showed aggravated behavioral changes after ROT injection without inducing cell apoptosis in other brain regions. The open field **(a-d)**, rotarod tests **(e)** and grip strength **(f)** were performed to evaluate locomotor activities and muscle strength. DDAH-1$^{-/-}$ rats' performance were worsened on rotating rods, open field test and grip strength as compared to DDAH-1$^{+/+}$ rats after ROT injection. (b) $F(3, 20) = 82.05$, $p < 0.0001$, (c) $F(3, 20) = 62.06$, $p < 0.0001$, (d) $F(3, 20) = 41.08$, $p < 0.0001$, (e) $F(3, 20) = 180.0$, $p < 0.0001$, (f) $F(3, 20) = 202.8$, $p < 0.0001$. **(g)** Quantitative analysis of

rotarod test showed alleviation of the motor deficit after L-DOPA/benserazide administration. $F(3, 20) = 20.37$, $p < 0.0001$. **(h)** Representative images of TUNEL assay in Ctx, Hip, CB and corpus callosum of DDAH-1$^{-/-}$ and WT rats after ROT injection. Results are presented as mean ± SD, n=6 rats/group. Statistical significance was determined using one-way ANOVA with Tukey post-hoc testing. Significance levels are indicated as n.s. $p > 0.05$, ** $p < 0.01$, *** $p < 0.001$. Abbreviations: ROT=rotenone, DDAH-1=dimethylarginine dimethylamino hydrolase-1, Ctx=cortex, Hip=hippocampus, CB=cerebellum, DAPI=2-(4-Amidinophenyl)-6-indolecarbamidine dihydrochloride, TUNEL=terminal deoxynucleotidyl transferase-mediated dUTP-biotin nick end labeling assay.

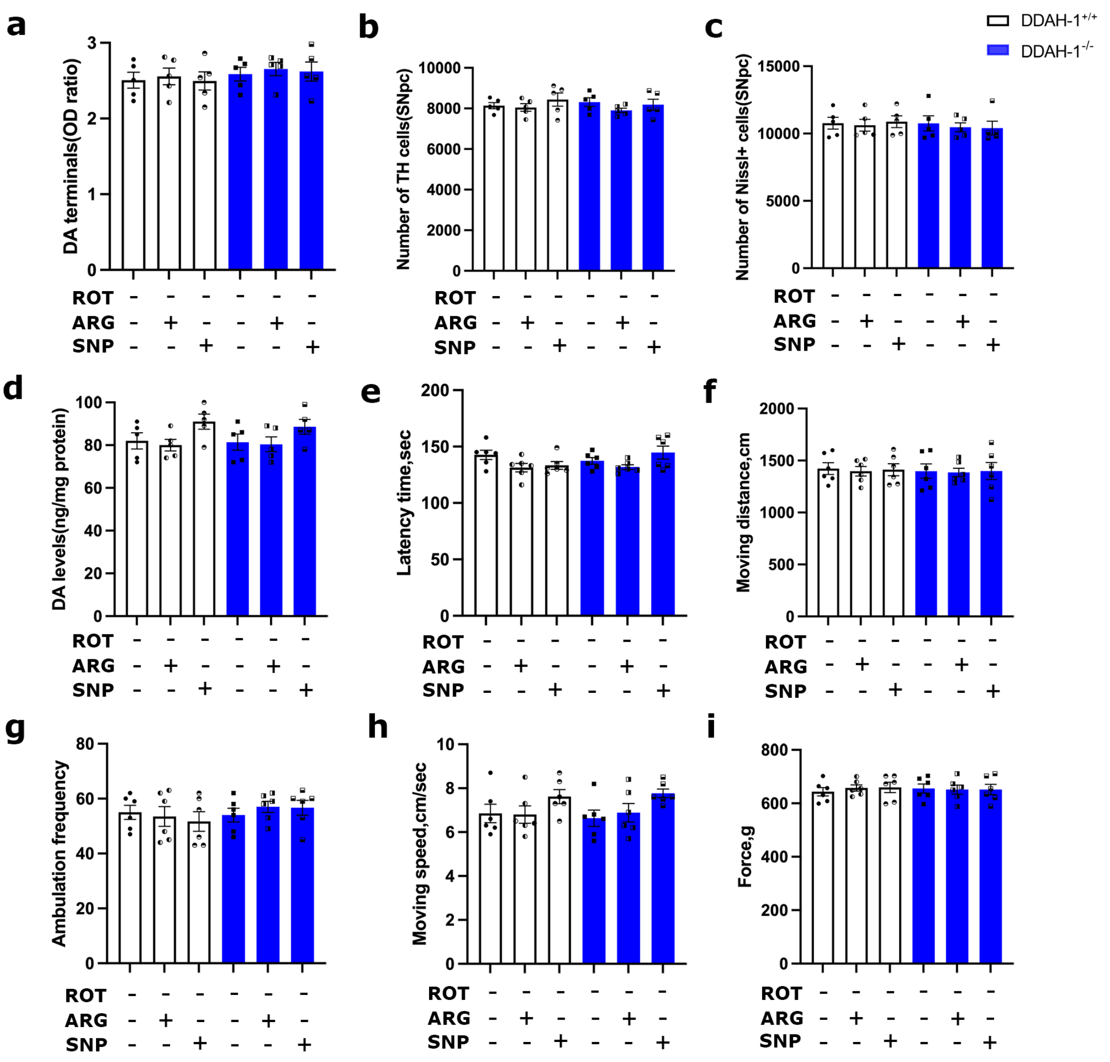

Fig.S3

The ARG regimen or NO donor administration did not cause dopaminergic neurons loss or behavioral alteration for rats with ROT injection. **(a-d)** ARG or SNP supplementing had no significant impact on the striatal dopamine terminals, TH-positive neurons levels in the SNpc or striatum dopamine levels for rats without ROT injection. (a) $F(5, 24) = 0.3373$, $p = 0.8852$, (b) $F(5, 24) = 0.7281$, $p = 0.6092$, (c) $F(5, 24) = 0.1605$, $p = 0.9746$, (d) $F(5, 24) = 1.828$, $p = 0.1453$. **(e-i)** Analysis on the open field, rotarod tests and grip strength showed ARG or SNP supplementing did not either lead to behavioral change for rats without ROT injection. (e) $F(5, 30) = 2.242$, $p = 0.0758$, (f) $F(5, 30) = 0.04683$, $p = 0.9986$, (g) $F(5, 30) = 0.4938$, $p = 0.7783$, (h) $F(5, 30) = 1.710$, $p = 0.1629$, (i) $F(5, 30) = 0.1163$, $p = 0.9878$. Results are presented as mean ± SD, n=5-6 rats/group. one-way ANOVA with Tukey post-hoc testing. Statistical significance was determined using one-way ANOVA with Tukey post-hoc testing. Significance levels are indicated as n.s. $p > 0.05$. Abbreviations: ROT=rotenone, ARG=L-arginine, SNP=sodium

nitroprusside, DDAH-1=dimethylarginine dimethylamino hydrolase-1, DA=dopamine, OD=optical density, TH=tyrosine hydroxylase, SNpc=substantia nigra pars compacta.

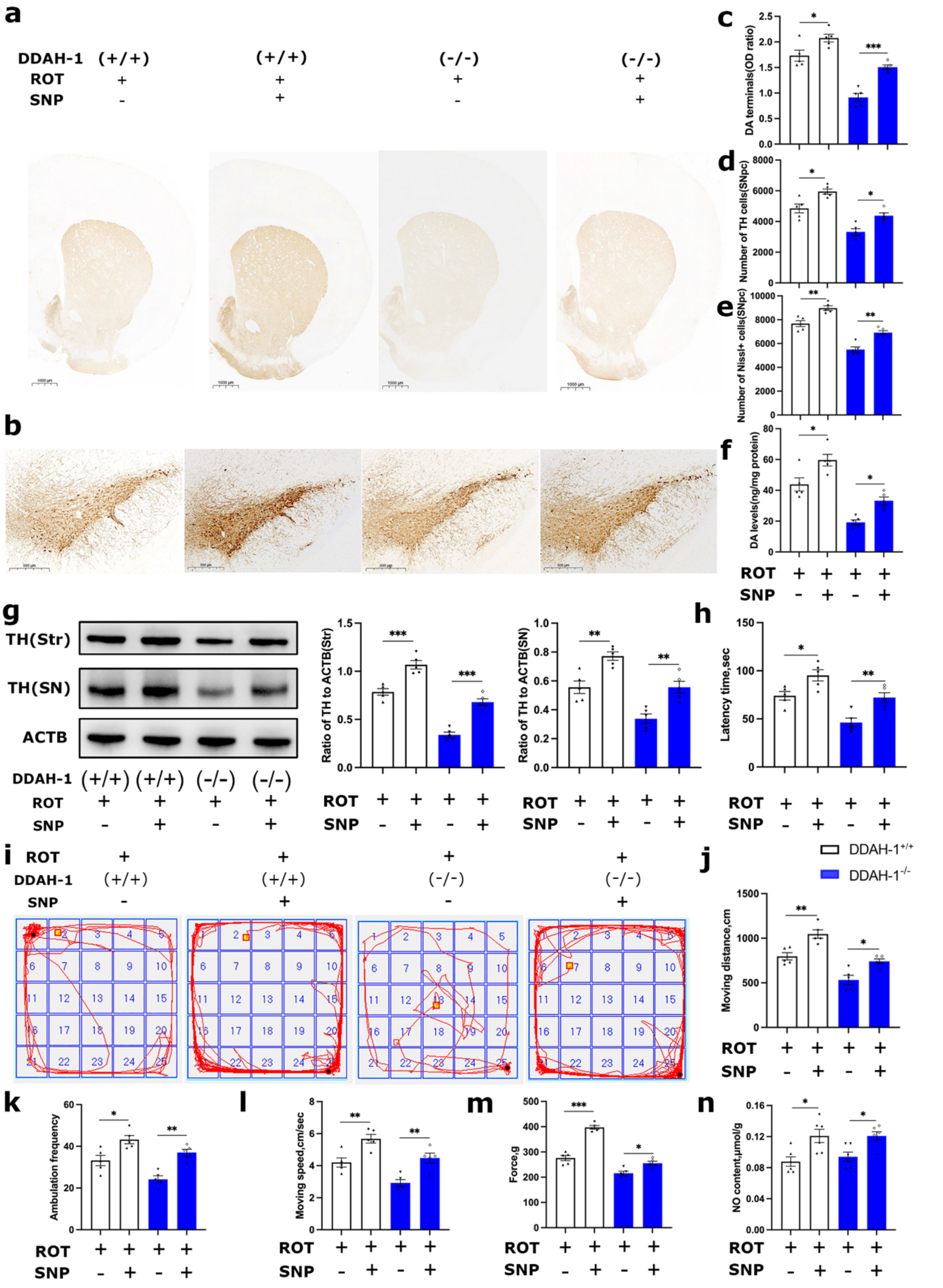

Fig. S4

Supplementing of NO donor to DDAH-1$^{-/-}$ rats alleviated ROT-induced dopaminergic neurodegeneration.

(a-b) Representative immunochemical staining images for TH-positive neurons in (a)Str and (b)SNpc.

(c-d) Striatal dopamine terminals and TH-positive neurons levels after SNP administration, including (c)the OD of dopamine terminals and (d)stereological cell counting of TH-positive neurons, which

showed a significant increase both in DDAH-$1^{+/+}$ and DDAH-$1^{-/-}$ rats after SNP supplement. (c) $F(3, 16) = 37.66$, $p < 0.0001$, (d) $F(3, 16) = 24.76$, $p < 0.0001$. **(e-f)** The number of Nissl-positive neurons in the SNpc and total striatum dopamine levels after SNP supplement. (e) $F(3, 16) = 47.59$, $p < 0.0001$, (f) $F(3, 16) = 28.34$, $p < 0.0001$. **(g)** Immunoblot and semi-quantitative analysis of TH expression in Str and SN after SNP treatment. (Str) $F(3, 16) = 73.12$, $p < 0.0001$, (SN) $F(3, 16) = 22.75$, $p < 0.0001$. **(h-m)** Quantitative analysis of rotarod test(h), open field(i-l) and grip strength(m) were performed to evaluate locomotor activities and muscle strength after SNP supplementing. (h) $F(3, 16) = 16.20$, $p < 0.0001$, (j) $F(3, 16) = 24.01$, $p < 0.0001$, (k) $F(3, 16) = 16.51$, $p < 0.0001$, (l) $F(3, 16) = 17.69$, $p < 0.0001$, (m) $F(3, 20) = 103.1$, $p < 0.0001$. **(n)** Quantitative analysis of NO concentrations in midbrain after SNP treatment. $F(3, 20) = 7.019$, $p = 0.0021$. Results are presented as mean ± SD, n=5-6 rats/group. Statistical significance was determined using one-way ANOVA with Tukey post-hoc testing. Significance levels are indicated as n.s. $p > 0.05$, * $p < 0.05$, ** $p < 0.01$, *** $p < 0.001$. Abbreviations: ROT=rotenone, SNP=sodium nitroprusside, NO=nitric oxide, DDAH-1=dimethylarginine dimethylamino hydrolase-1, DA=dopamine, OD=optical density, TH=tyrosine hydroxylase, SNpc=substantia nigra pars compacta, SN=substantia nigra, Str=striatum, ACTB=β-actin.

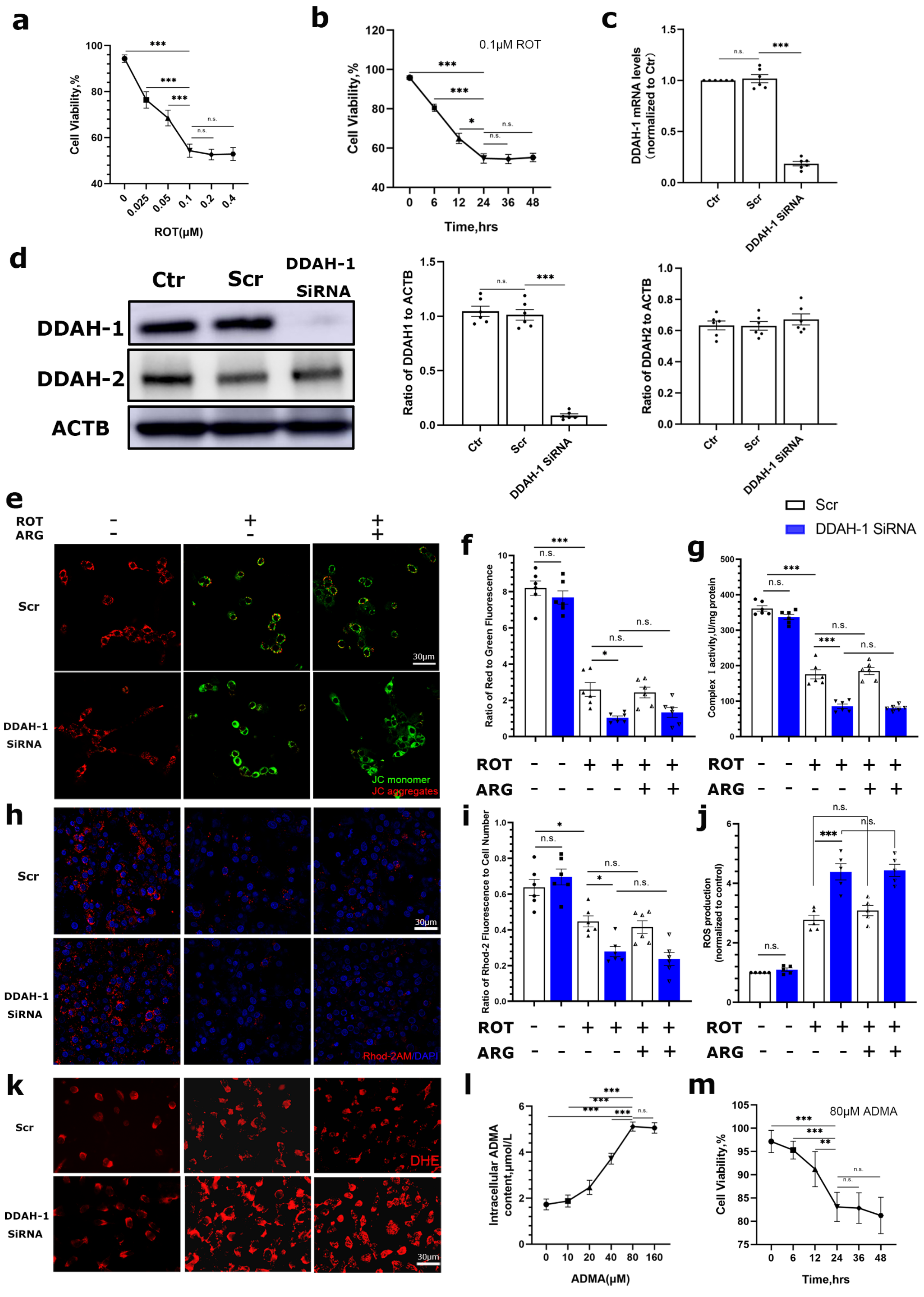

Fig. S5

Exploration of experiment condition and the DDAH-1 siRNA effects on PC12 cells. **(a)** Quantifications of the cytotoxicity of ROT in different concentrations. $F(5, 24) = 174.4$, $p < 0.0001$. **(b)** Quantifications of the cytotoxicity of 0.1 μM ROT at different time points. $F(5, 24) = 65.83$, $p < 0.0001$. **(c)** The semi-quantitative analysis of DDAH-1 mRNA levels with DDAH-1 siRNA transfection. $F(2, 15) = 332.7$, $p <$

0.0001. **(d)** Representative immunoblot of protein of DDAH-1 and DDAH-2 the semi-quantitative analysis with DDAH-1 siRNA transfection. DDAH-1: $F(2, 15) = 186.3$, $p < 0.0001$. DDAH-2: $F(2, 15) = 0.5675$, $p = 0.5786$. **(e-f)** Representative images and quantitative analysis of mitochondrial membrane potential (MMP) measurement by JC-1 staining. Red aggregates indicate normal MMP while green monomers indicate disrupted MMP. $F(5, 30) = 101.6$, $p < 0.0001$. **(g)** Quantitative analysis of MRCC I activity. $F(5, 30) = 203.7$, $p < 0.0001$. **(h-i)** Representative images of mitochondrial Ca^{2+} displayed by red Rhod-2AM staining and its quantitative analysis. Rhod-2 fluorescence normalized by DAPI-positive cells. $F(5, 30) = 24.83$, $p < 0.0001$. **(j-k)** Representative images of DHE staining for ROS measurement and statistical analysis of ROS fluorescence intensity normalized to the control. $F(5, 24) = 77.84$, $p < 0.0001$. **(l)** Quantifications of the cytotoxicity of ADMA in different concentrations. $F(5, 24) = 192.4$, $p < 0.0001$. **(m)** Quantifications of the cytotoxicity of 80 μM ADMA at various time points. $F(5, 24) = 24.55$, $p < 0.0001$. Results are presented as mean ± SD, n=5-6 independent experiments. Statistical significance was determined using one-way ANOVA with Tukey post-hoc testing. Significance levels are indicated as n.s. $p > 0.05$, * $p < 0.05$, ** $p < 0.01$, *** $p < 0.001$. Abbreviations: ROT=rotenone, ADMA=asymmetric dimethylarginine, DDAH-1=dimethylarginine dimethylamino hydrolase-1, DDAH-2=dimethylarginine dimethylamino hydrolase-2, ARG=L-arginine, ACTB=β-actin, siRNA=small interfering RNA, Scr=scramble, ROS=reactive oxygen species, DHE=dihydroethidium.

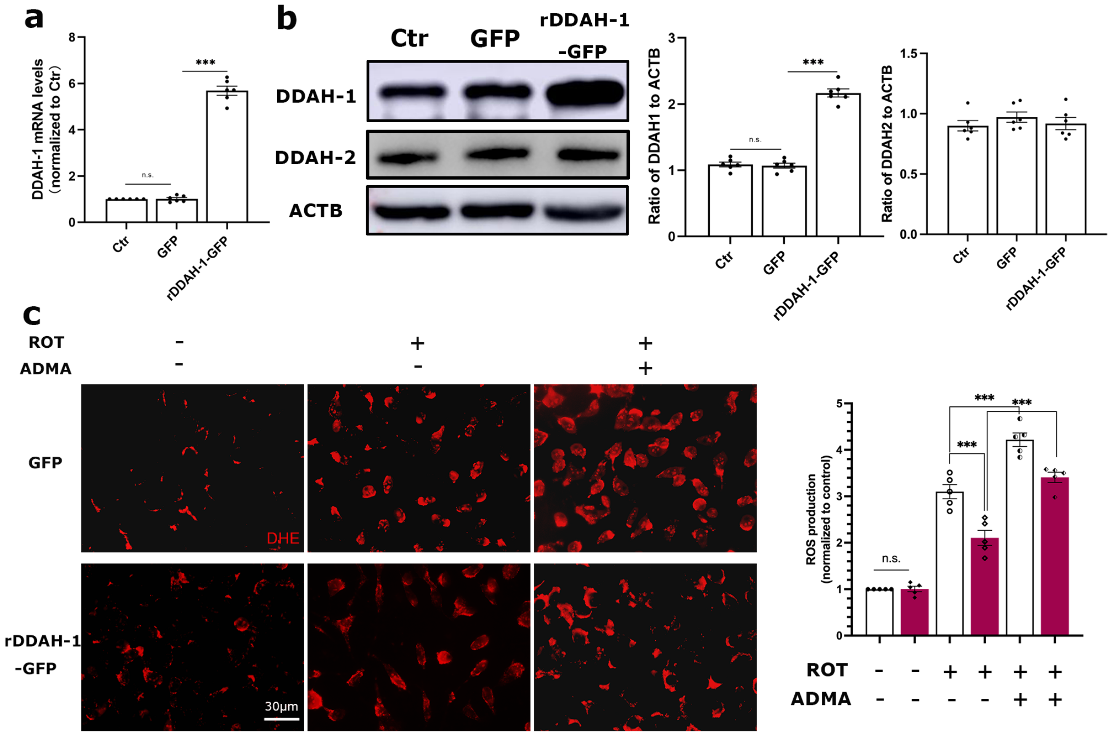

Fig. S6

DDAH-1 overexpression could alleviate ROT-induced ROS production by degrading ADMA. **(a-b)** The semi-quantitative analysis of DDAH-1 mRNA and protein levels of DDAH-1 and DDAH-2 with DDAH-1 rDDAH-1 plasmid transfection. (a) $F(2, 15) = 524.6$, $p < 0.0001$, (b) DDAH-1: $F(2, 15) = 185.6$, $p < 0.0001$, DDAH-2: $F(2, 15) = 0.6766$, $p = 0.5232$. **(c)** Representative images of DHE staining for ROS measurement and statistical analysis of ROS fluorescence intensity normalized to the control. $F(5, 24) = 122.5$, $p < 0.0001$. Results are presented as mean ± SD, n=5-6 independent experiments. Statistical significance was determined using one-way ANOVA with Tukey post-hoc testing. Significance levels are indicated as n.s. $p > 0.05$, *** $p < 0.001$. Abbreviations: ROT=rotenone, ADMA=asymmetric dimethylarginine, DDAH-1=dimethylarginine dimethylamino hydrolase-1, DDAH-2=dimethylarginine dimethylamino hydrolase-2, ACTB=β-actin, GFP=green-fluorescent protein, Ctr=control, ROS=reactive oxygen species, DHE=dihydroethidium.

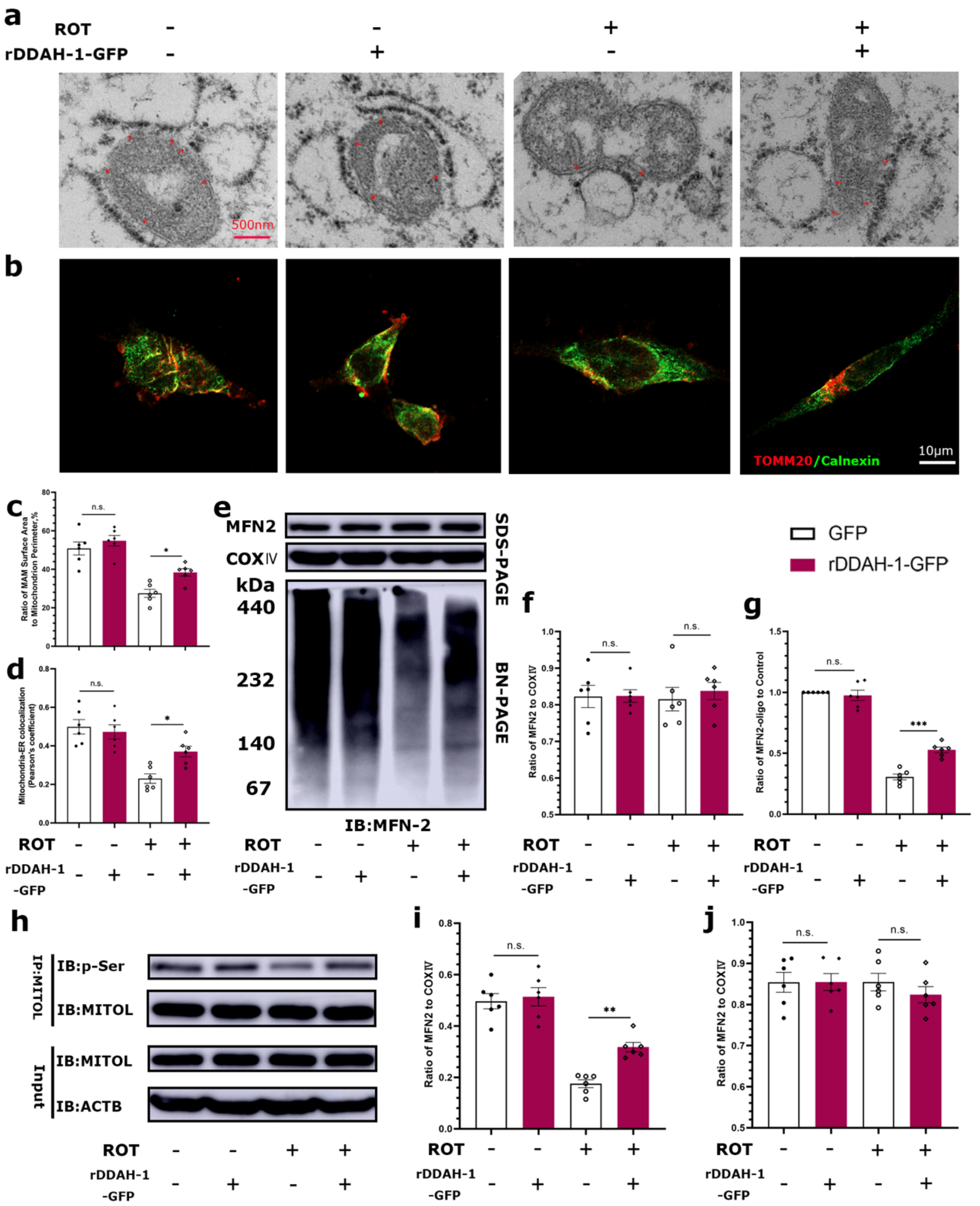

Fig. S7

DDAH-1 overexpression prevented ROT-induced disruptions of ER-mitochondrial contacts and MFN2 oligomerization. **(a)** Representative TEM images of the morphology of ER-mitochondrial contacts. Red arrows indicated the ER-mitochondrial surface area. **(b)** Representative images of co-immunofluorescence staining for TOMM20 and calnexin. **(c)** Quantitation of ER length adjacent to mitochondria normalized by mitochondrial perimeter. F(3, 20) = 23.15, $p < 0.0001$. **(d)** Quantitation of mitochondrial-ER colocalization. F(3, 20) = 14.41, $p < 0.0001$. **(e-g)** The steady-state levels and

oligomerization of MFN2 were determined in isolated mitochondria from PC12 cells using BN-PAGE and immunoblotting. Treatment of ROT or rDDAH-1-GFP transfection did not change the steady-state levels of MFN2, while DDAH-1 overexpression alleviated the reduction of MFN2 oligomerization induced by ROT. (f) $F(3, 20) = 0.1241$, $p = 0.9448$, (g) $F(3, 20) = 164.2$, $p < 0.0001$. **(h-j)** Total cell lysates were immunoprecipitated with anti-MITOL and immunoblotted with p-Ser antibodies. (h)Representative immunoprecipitation/immunoblot of phosphorylated MITOL. (i-j) Quantitive data represent that DDAH-1 overexpression alleviated ROT-induced decrease of the phosphorylation of MITOL at serine. (i) $F(3, 20) = 38.10$, $p < 0.0001$, (j) $F(3, 20) = 0.5056$, $p = 0.6828$. Results are presented as mean ± SD, n=6 independent experiments. Statistical significance was determined using one-way ANOVA with Tukey post-hoc testing. Significance levels are indicated as n.s. $p > 0.05$, * $p < 0.05$, ** $p < 0.01$, *** $p < 0.001$. Abbreviations: ROT=rotenone, DDAH-1=dimethylarginine dimethylamino hydrolase-1, TOMM20=translocase of outer mitochondrial membrane 20 homolog, MFN2=mitofusin2, COXIV=cytochrome c oxidase IV, siRNA=small interfering RNA, GFP=green-fluorescent protein, MAM=mitochondria-associated endoplasmic reticulum membrane, ER=endoplasmic reticulum, ACTB=β-actin, MITOL=mitochondrial ubiquitin ligase.
