# Supplementary material for: DDAH-1 maintains endoplasmic reticulum-mitochondria contacts and protects dopaminergic neurons in Parkinson’s disease
Source: Cell Death Dis. 2024 Jun 7;15(6):399. doi: 10.1038/s41419-024-06772-w (PMC11161642; doi:10.1038/s41419-024-06772-w)
Supplement: Supplementary file 2 — Full and uncropped western blots [file 41419_2024_6772_MOESM2_ESM.pdf]

Fig. 1c

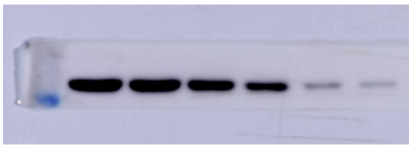

DDAH-1

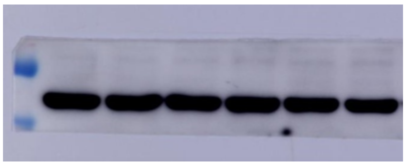

ACTB

Fig. 2g

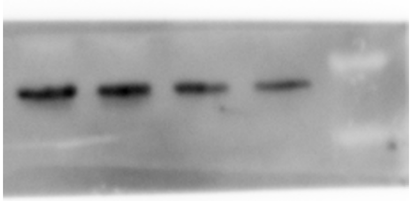

TH(Str)

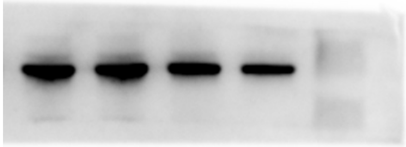

TH(SN)

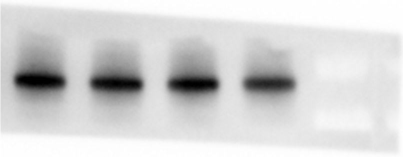

ACTB

Fig. 3g

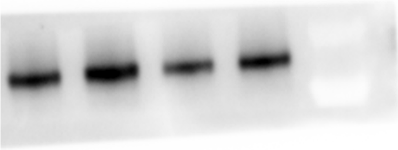

TH(Str)

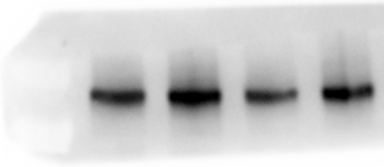

TH(SN)

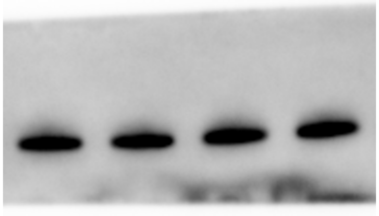

ACTB

Fig. 6e

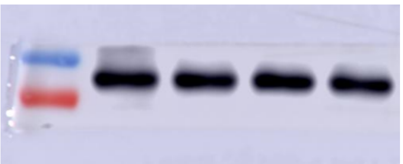

Total-MFN2

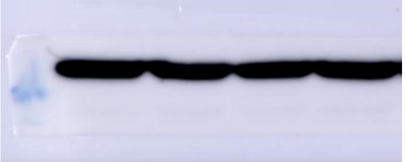

COX IV

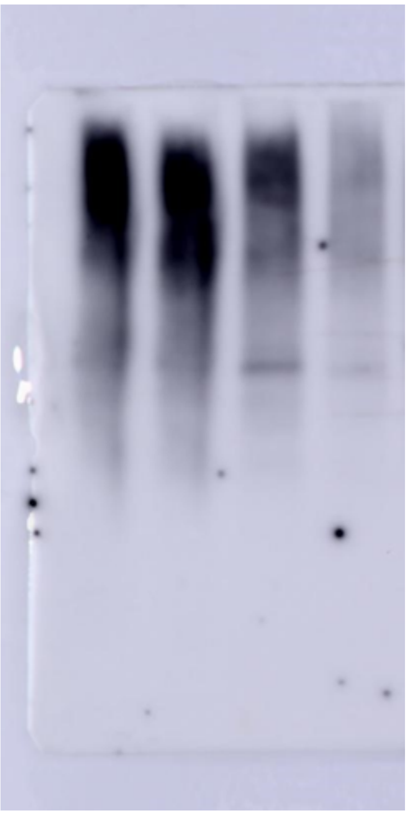

Oligo-MFN2

Fig. 6h

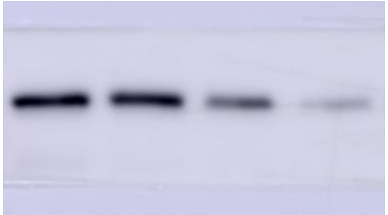
p-Ser

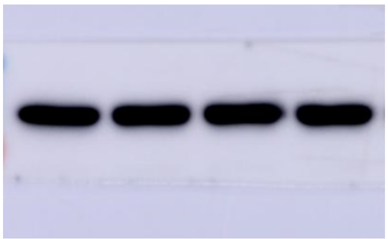
IP：MITOL

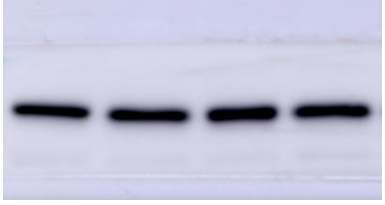
Input-MITOL

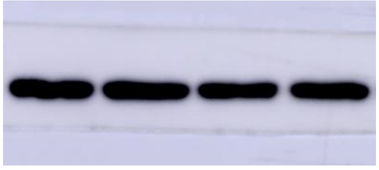
Input-ACTB

Fig. S4g

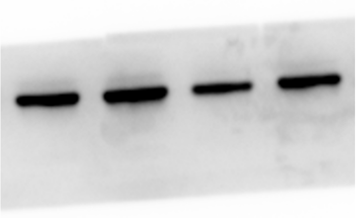
TH(Str)

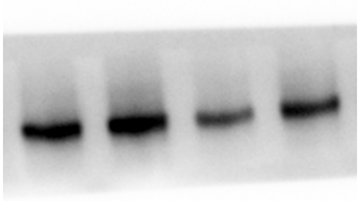
TH(SN)

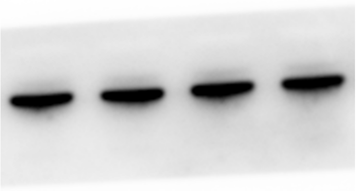
ACTB

Fig. S5d

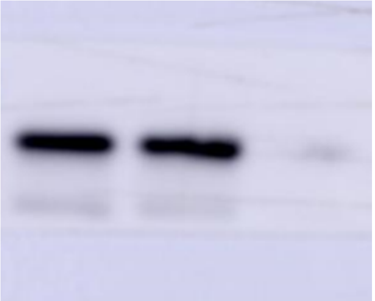

DDAH-1

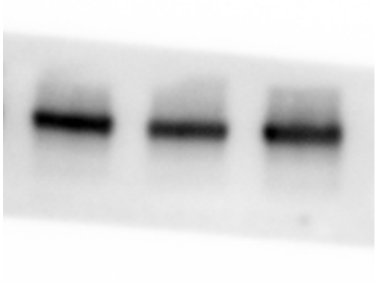

DDAH-2

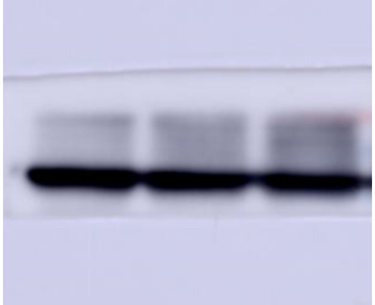

ACTB

Fig. S6b

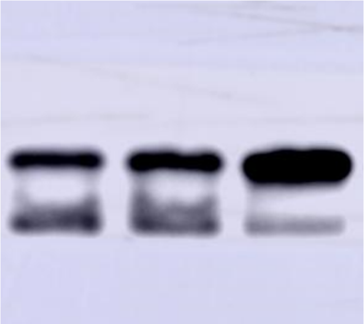

DDAH-1

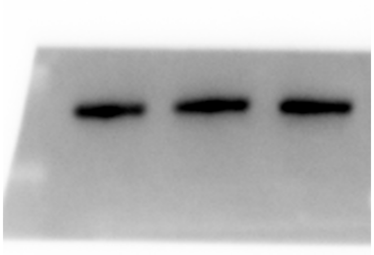

DDAH-2

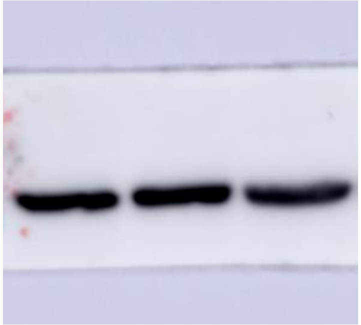

ACTB

Fig. S7e

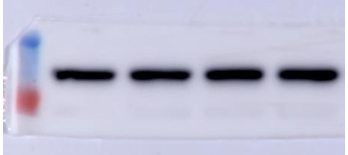

Total-MFN2

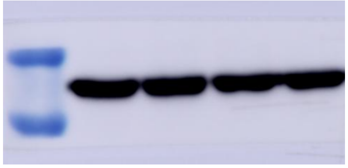

COX IV

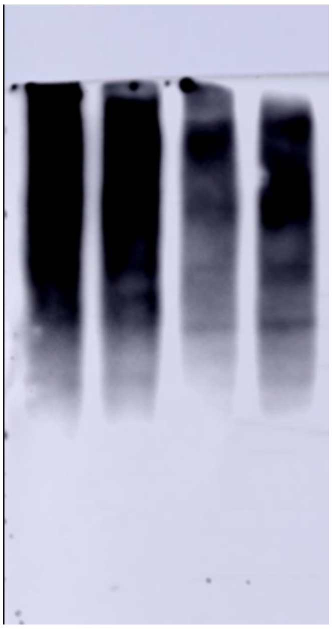

Oligo-MFN2

Fig. S7h

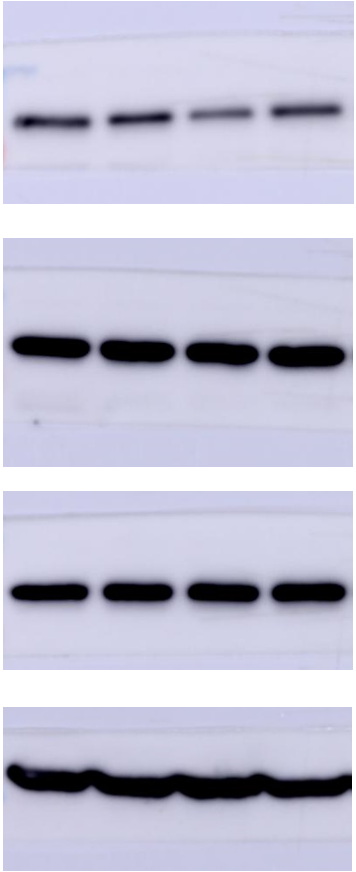

p-Ser

IP：MITOL

Input-MITOL

Input-ACTB
